# Mapping malignant T‐cell states and immune circuits in Sézary syndrome by single‐cell analysis

**DOI:** 10.1111/jdv.70368

**Published:** 2026-03-03

**Authors:** Beth A. Childs, Eslam A. Elghonaimy, Praveen Ramakrishnan Geethakumari, Kiran A. Kumar, Joseph F. Merola, Heather W. Goff, Todd A. Aguilera

**Affiliations:** ^1^ Department of Radiation Oncology UT Southwestern Dallas Texas USA; ^2^ Department of Dermatology UT Southwestern Dallas Texas USA; ^3^ Division of Hematology Oncology, Department of Medicine UT Southwestern Dallas Texas USA; ^4^ Division of Rheumatology, Department of Medicine UT Southwestern Dallas Texas USA

**Keywords:** cell communication, cutaneous T‐cell lymphoma, immune evasion, Sézary syndrome, single‐cell RNA sequencing, T‐lymphocytes

## Abstract

**Background:**

Sézary syndrome (SS) and leukaemic mycosis fungoides (MF) are aggressive cutaneous T‐cell lymphomas (CTCLs) with poor prognosis and limited treatment options. Single‐cell RNA sequencing (scRNA‐seq) offers an opportunity to dissect malignant heterogeneity and immune dysregulation yet has been underutilized in leukaemic CTCL.

**Objectives:**

To define transcriptional heterogeneity within malignant T cells, identify immunomodulatory signals shaping the tumour microenvironment and uncover actionable vulnerabilities in SS and leukaemic MF.

**Methods:**

We analysed 144,321 peripheral blood mononuclear cells from 22 patients (SS or leukaemic MF) and 7 healthy controls using scRNA‐seq, with CITE‐seq and TCR‐seq in subsets. Public and prospectively collected datasets were integrated after rigorous per‐sample quality control, batch correction and malignancy assignment. Differential gene expression and cell–cell communication analyses were performed using complementary modelling approaches with correction for sparsity and multiple testing.

**Results:**

Three transcriptionally distinct malignant T‐cell (MTC) subtypes were identified: central memory‐like (MTC CM), effector/effector memory‐like (MTC E/EM) and regulatory‐like (MTC Reg), each with unique expression programmes and putative vulnerabilities. MTC CM, the dominant population, expressed central memory markers, Th2/Th22 skewing and a broader KIR repertoire (*KIR3DL2, KIR3DL1, KIR2DL3*) than previously reported. MTC E/EM and MTC Reg were enriched for *NR4A1* and *ITGB1*, respectively. Cell–cell communication analysis revealed KIR–MHC‐I signalling and suggested that MTC‐derived TNF and IL‐10 may drive JAK/STAT pathway activity in non‐T cells, indicating an underrecognized dimension of tumour‐induced immune reprogramming.

**Conclusions:**

This is one of the most comprehensive single‐cell studies of leukaemic CTCL to date. We uncover new malignant T‐cell states, broaden the known repertoire of KIR expression, and propose mechanisms of immune evasion that may contribute to treatment resistance. These findings lay the groundwork for subtype‐specific and microenvironment‐informed therapeutic strategies in Sézary syndrome, with potential implications for guiding targeted therapy selection but require validation in larger, independent cohorts.

## Plain Language Summary

Sézary syndrome is a rare and aggressive blood cancer that affects certain immune cells called T cells. It causes widespread red, itchy skin and affects fewer than 1 in 1 million people. The disease is difficult to treat and often resistant to therapy.

This study was conducted in Dallas, TX, United States, with additional data from previously published studies. The goal was to better understand Sézary syndrome by analysing individual immune cells from the blood of affected patients. Researchers used a high‐resolution technique called single‐cell RNA sequencing, which allows scientists to study thousands of individual cells at once and identify differences between them.

The study examined more than 140,000 immune cells from 22 patients and 7 healthy individuals. The team discovered that the cancerous T cells could be grouped into three distinct subtypes, each with different behaviours and potential drug targets. One subtype showed signs of immune suppression; another appeared more vulnerable to energy and metabolic‐related interventions; and another appeared more vulnerable to cell death. The researchers also found that cancer cells interacted with nearby immune cells in ways that may help the cancer evade treatment and weaken the body’s natural defences.

These findings help explain why Sézary syndrome is so hard to treat and highlight new directions for more personalized therapies. Understanding the different types of cancer cells in each patient could eventually lead to better‐targeted treatments and improved outcomes for people living with this challenging disease.

## Graphical abstract

Peripheral blood single‐cell RNA‐seq from leukaemic CTCL defined three malignant T‐cell programmes: MTC CM, MTC Reg and MTC E/EM, each with distinct features and candidate vulnerabilities. For example, inferred immune circuits highlighted actionable IL‐10/JAK–TYK2–STAT3 signalling, KIR–MHC I inhibitory interactions and myeloid/B‐cell inflammatory and antigen presentation programmes. Created in BioRender. Childs, B. (2025) https://BioRender.com/7cmpnop

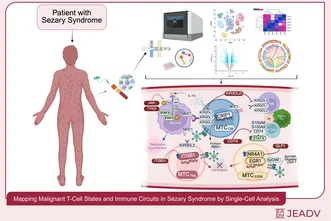

**Why was the study undertaken?**

- To better understand the cellular heterogeneity and immune evasion mechanisms in Sézary syndrome, a rare and aggressive cutaneous T‐cell lymphoma, by applying single‐cell RNA sequencing to peripheral blood samples from affected patients.

**What does the study add?**

- This study identifies three transcriptionally distinct malignant T‐cell subtypes with unique therapeutic vulnerabilities in Sézary syndrome and leukaemic mycosis fungoides. It uncovers novel KIR–MHC‐I interactions involving malignant T cells and surrounding immune cells. Notably, it shows that malignant T‐cell–derived cytokines, including IL‐10 and TNF, drive JAK/STAT pathway activation in myeloid cells rather than T cells, suggesting a mechanism by which malignant cells may reprogramme the immune microenvironment to promote immune evasion.

**What are the implications of this study for disease understanding and/or clinical care?**

- These findings highlight biologically distinct malignant T‐cell states and immune environment crosstalk that could inform selection of targeted therapies (e.g., KIR blockade, integrin modulation, cytokine/JAK–STAT‐directed approaches) and guide more individualized treatment strategies in Sézary syndrome.

## INTRODUCTION

Sézary syndrome (SS) and leukaemic variants of mycosis fungoides (MF) are aggressive subtypes of cutaneous T‐cell lymphoma (CTCL) characterized by clonal expansion of skin‐homing T cells and profound immune dysfunction. Once significant blood involvement develops, median survival decreases to 1–4 years.^1^ Despite recent advancements in diagnostic technologies, such as immunophenotyping and T‐cell receptor (TCR) clonality analyses, definitive classification remains elusive. Common immunophenotypic markers—including CD4+CD26− and CD4+CD7− subsets—lack the sensitivity and specificity required for accurate disease stratification.^2^

The cellular origins, transcriptional programmes and mechanisms of immune escape in leukaemic CTCL remain incompletely understood, limiting progress in targeted therapy.^3^, ^4^ Single‐cell RNA sequencing (scRNA‐seq) now offers the ability to resolve malignant T‐cell (MTC) heterogeneity and characterize immune dysregulation at high resolution.

**FIGURE 1:**
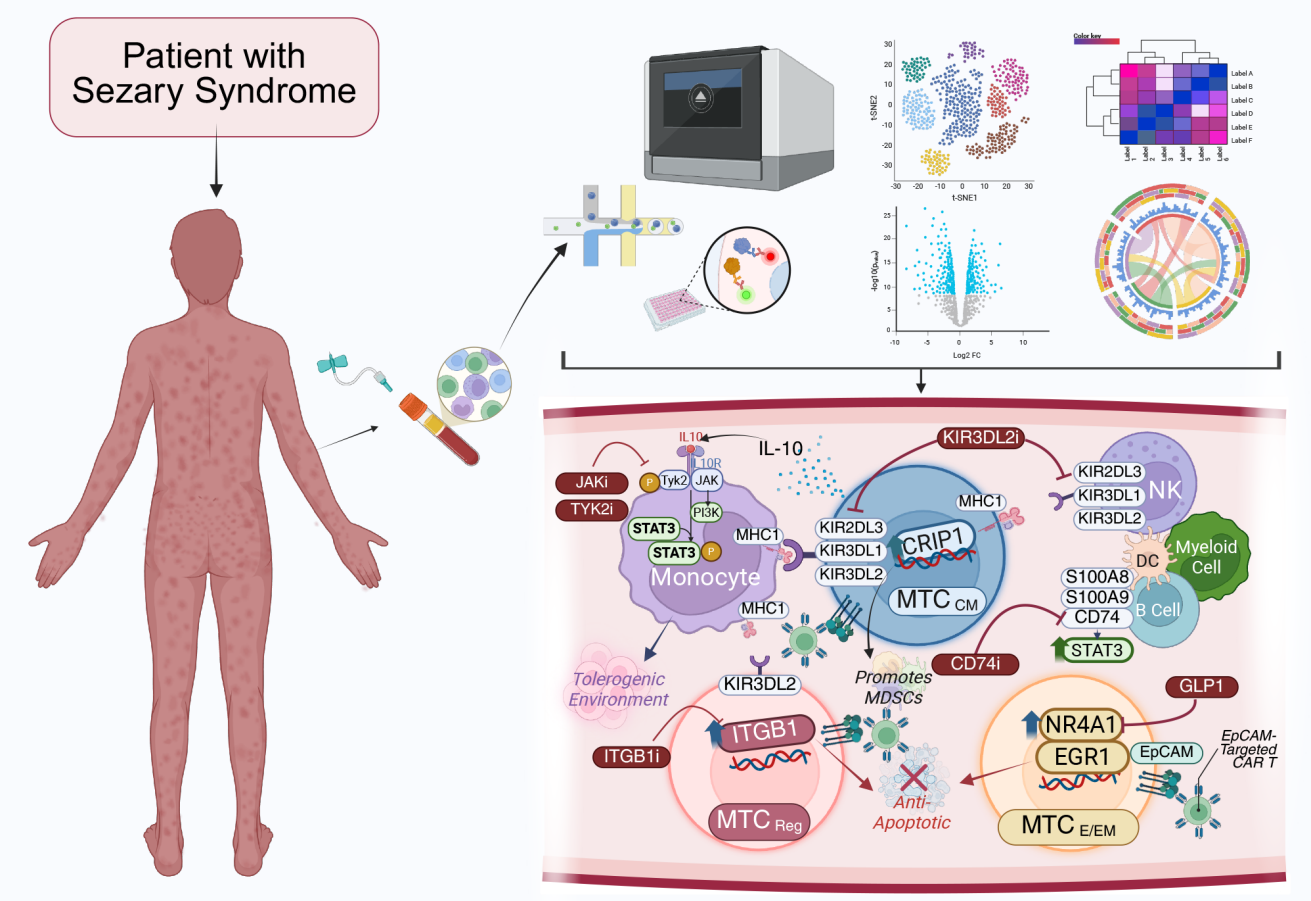
Mapping malignant and immune landscapes in Sézary syndrome via single‐cell multiomics. Single‐cell profiling of peripheral blood from Sézary syndrome patients reveals three malignant T‐cell subtypes and identifies immune interactions that may promote immune evasion. Findings highlight both subtype‐specific vulnerabilities and broader mechanisms of immune suppression that could inform future therapeutic strategies. Created in BioRender. Childs, B. (2025) https://BioRender.com/inu4tgl.

Here, we employed a high‐resolution approach based on scRNA‐seq to clarify the tumour and peripheral immune landscape. We evaluated peripheral blood mononuclear cells (PBMCs) from 22 leukaemic CTCL patients and 7 healthy controls (HCs), then integrated these data with paired TCR sequencing (TCR‐seq) and Cellular Indexing of Transcriptomes and Epitopes by Sequencing (CITE‐seq) proteomic profiling.^2^, ^5^, ^6^, ^7^ We identify previously unrecognized MTC transcriptional states and uncover immune evasion programmes, including upregulation of inhibitory killer cell immunoglobulin‐like receptors (KIRs) beyond *KIR3DL2* on malignant and non‐malignant cells. Additionally, while prior studies have highlighted JAK/STAT in malignant SS cells, our data unexpectedly show stronger STAT3 activation in myeloid populations, driven by inflammatory signalling through *S100A8/A9* and *CD74*. Finally, we explore subtype‐specific vulnerabilities that may inform future therapies. Our findings propose immune evasion mechanisms and nominate both subset‐specific and broader therapeutic strategies to guide targeted treatment in leukaemic CTCL (Figure 1).

## MATERIALS AND METHODS

Please refer to Supplemental Methods in Appendix S1.

## RESULTS

### Project design and cell annotations

**FIGURE 2:**
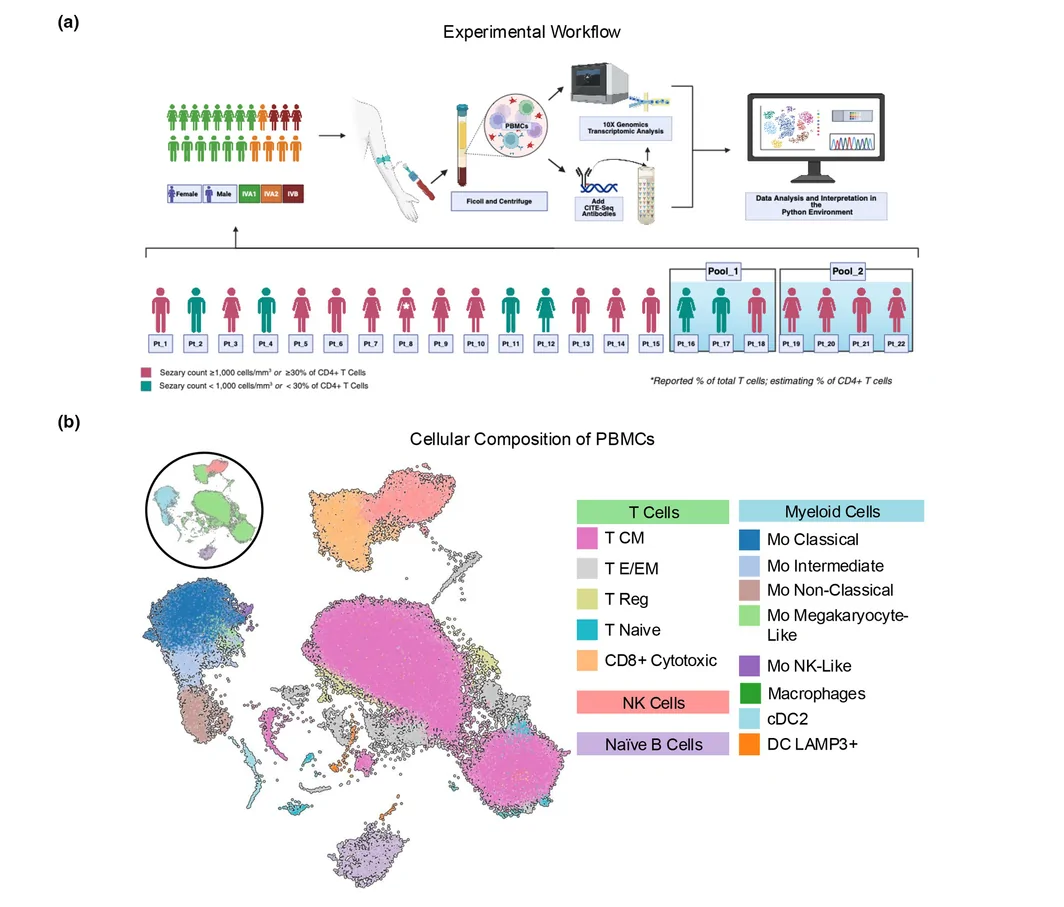
Project workflow and cellular composition in CTCL. (a) Overview of single‐cell RNA sequencing (scRNA‐seq) analysis pipeline, encompassing pre‐processing, quality control and Harmony‐based integration of 101,710 peripheral blood mononuclear cells (PBMCs) from 22 patients with cutaneous T‐cell lymphoma (CTCL). Downstream analyses included 70,519 high‐quality cells. (b) UMAP visualization of all PBMCs, coloured by major immune lineages and refined subsets. Broad populations include T cells, natural killer (NK) cells, naïve B cells and myeloid cells. Further subclustering identified distinct T‐cell phenotypes (e.g., central memory [CM], effector/memory [E/EM], regulatory [Reg], naïve and cytotoxic) and diverse myeloid subsets, including classical, intermediate and non‐classical monocytes (Mo), Mo megakaryocyte‐like, Mo NK‐like, type 2 conventional dendritic cells (cDC2s), macrophages (MP) and LAMP3+ dendritic cells (DCs).

To characterize the immune landscape of CTCL, we analysed scRNA‐seq data from 144,321 PBMCs, including 101,710 cells from 22 patients with SS or leukaemic MF (8 from our institution and 14 from public datasets) and 42,611 cells from seven HCs (Figure 2a).^5^, ^6^, ^7^, ^8^, ^9^ CTCL patients (mean age 67; 12 women, 10 men) exhibited variable clinical courses (Table 1, Table S1). Following quality control (QC) and integration, 70,519 cells from CTCL and 37,100 from HCs were retained (Figure S1A–C).

**TABLE 1:** Patient demographics and clinical characteristics.

Pool/group	Sample	Age range (years)	Sex	Sezary count (cells/mm^3^)/percentage of CD4^+^ T cells	Prior treatments	Treatment at time of sampling	Clinical description
	Pt_1	70–79	M	31%	Mogamulizumab	NBUVB, Romidepsin	Erythroderma, >90% BSA with patches and plaques
	Pt_2	60–69	M	893	Bexarotene, IFNa	Vorinostat, ECP	Not available
	Pt_3	70–79	F	6129	MTX	Bexarotene	Not available
	Pt_4	60–69	M	110	NA	IFNa, ECP	Not available
	Pt_5	80–89	F	1968	NBUVB	ECP	Not available
	Pt_6	70–79	M	1200	Pred	ECP, bexarotene	Not available
	Pt_7	80–89	F	6300	NA	Romidepsin	Not available
	Pt_8*	60–69	F	13%*	Bexarotene, TSEB, gemcitabine	7‐year no treatment	Generalized patches, plaques and tumours
	Pt_9	30–39	F	36%	MTX, IFNa, Romidepsin	None for 2 weeks	Plaques
	Pt_10	40–49	F	60%	NA	NA	<1% TBSA patches
	Pt_11	60–69	M	17%	NA	NA	Erythroderma
	Pt_12	70–79	F	21%	NA	NA	Plaques/rashes
	Pt_13	50–59	M	36%	NA	NA	Erythroderma
	Pt_14	70–79	F	60%	NA	ECP	85% TBSA, erythroderma
	Pt_15	70–79	M	46%	NA	NA	100% TBSA, erythroderma
Pool_1	Pt_16	70–79	F	9%	ECP, IFNa	IFNa	Patches and plaques
	Pt_17	50–59	M	67%	CysA	Romidepsin	Erythroderma, keratoderma
	Pt_18	50–59	M	92%	MMF, adalimumab, ECP	IFNa	Erythroderma, keratoderma
Pool_2	Pt_19	80–89	F	85%	NA	NA	Erythroderma, keratoderma
	Pt_20	70–79	F	73%	NA	Acitretin	Patches and thin plaques on trunk, buttocks and extremities
	Pt_21	40–49	M	76%	Acitretin, INFa, ECP, Romidepsin	Bexarotene	Erythroderma, keratoderma
	Pt_22	70–79	F	98%	IFN, ECP, Bexarotene, Acitretin, NBUVB	Romidepsin	Erythroderma
Healthy control	HC_1	50–59	F	NA	NA	NA	HLD, bipolar/depression
	HC_2	60–69	F	NA	NA	NA	DVT, depression, recent flu
	HC_3	60–69	F	NA	NA	NA	Schizophrenia, seizures
	HC_4	50–59	M	NA	NA	NA	Asthma
	HC_5	30–39	M	NA	NA	NA	NA
	HC_6	50–59	M	NA	NA	NA	HLD, lung disease
	HC_7	40–49	M	NA	NA	NA	NA

*Note*: This table summarizes the demographics and clinical characteristics of the patient cohort analysed in this study. It includes the sample identifiers grouped by study pool, clinical diagnoses, range encompassing patient age in years, sex, Sézary cell count expressed as absolute cells per cubic millimetre or as a percentage of total CD4+ T cells (*percentage provided as total T cells in study GSE165623), prior treatments and treatments administered within 2 weeks of sample collection (only systemic and lymphoma‐specific therapies listed). The final column provides a brief clinical description for patients diagnosed with cutaneous T‐cell lymphoma (CTCL) or lists other known diagnoses for healthy control (HC) participants.

Abbreviations: BSA, body surface area; CysA, cyclosporine A; DVT, deep vein thrombosis; ECP, extracorporeal photopheresis; HC, healthy control; HLD, hyperlipidaemia; IFNa, interferon‐alpha; MMF, mycophenolate mofetil; MTX, methotrexate; NA, not available; NBUVB, narrowband ultraviolet B phototherapy; Pred, prednisone; TBSA, total body surface area; TSEB, total skin electron beam radiation therapy.

Unsupervised clustering revealed major immune lineages, including T cells, myeloid cells, natural killer (NK) cells and B cells. Subclustering identified canonical T‐cell subsets, along with diverse monocyte and dendritic cell (DC) populations, naïve B cells and natural killer (NK) cells (Figure 2b, Figure S1D–G, Tables S2–S4).^10^, ^11^, ^12^

### Heterogeneity of malignant T cells reveals three dominant subtypes

**FIGURE 3:**
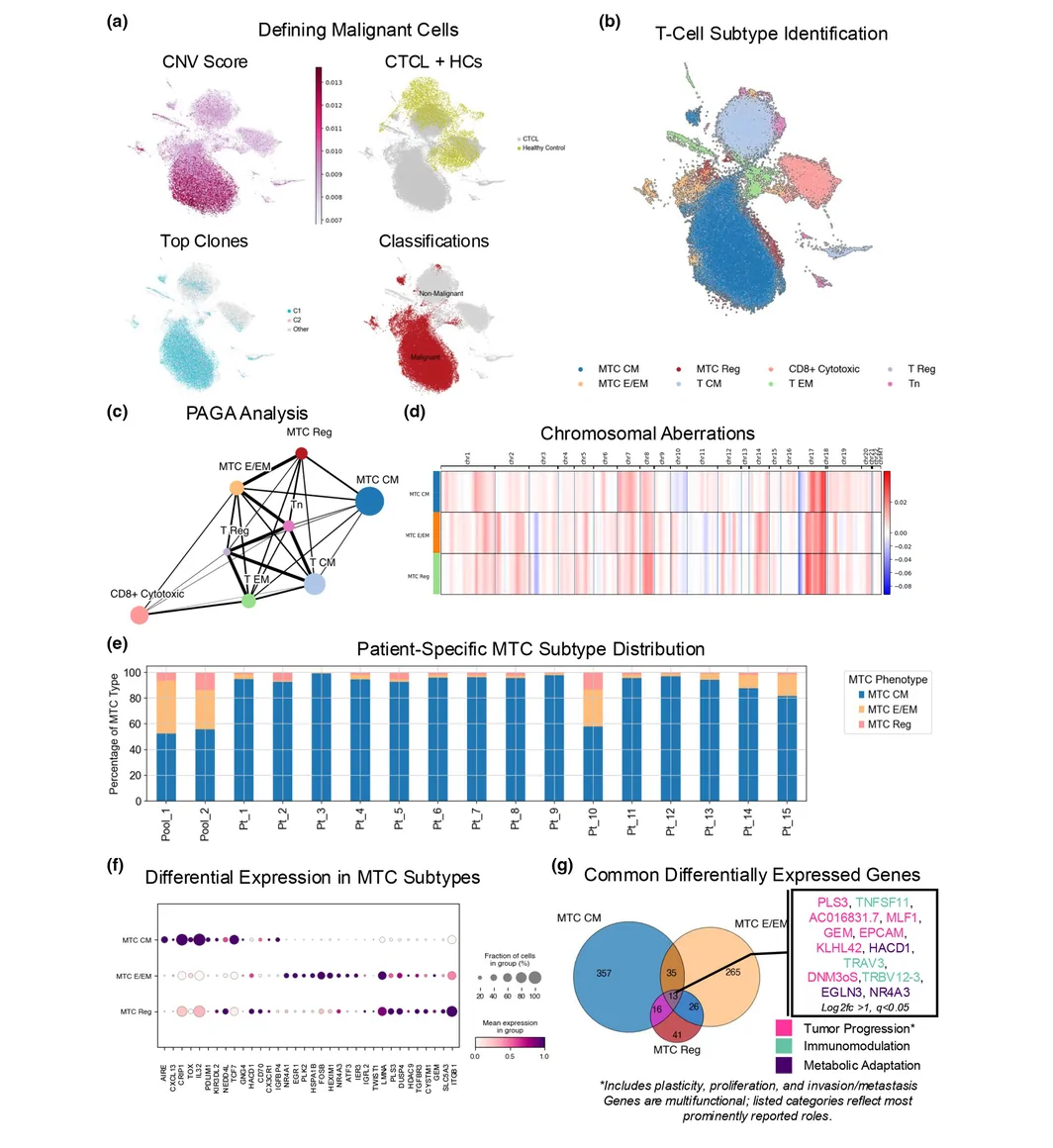
Diverse malignant T‐cell (MTC) subtypes reveal distinct pathogenic and therapeutic signatures. (a) Panels illustrating malignancy criteria including copy number variation (CNV) scores, comparative visualization with healthy controls, malignant versus benign T‐cell labelling and top clonotype frequency based on T‐cell receptor (TCR) sequencing data. (b) UMAP visualization of T‐cell clusters in CTCL patients, identifying distinct MTC subtypes: MTC CM (central memory‐like, Th2/Th22‐skewed), MTC E/EM (effector/effector memory, Th1‐biased) and MTC Reg (regulatory/exhausted‐like). (c) Plot illustrating connectivity and potential transitional relationships between malignant and benign T‐cell subsets, suggesting possible plasticity between MTC E/EM and MTC Reg. (d) Heatmap summarizing distinct CNV patterns across MTC subtypes, generated with inferCNVpy. (e) Bar graph illustrating the variability in MTC subtype composition across individual patient samples, demonstrating that MTC CM is the dominant MTC state (>90% of all MTCs), with smaller MTC E/EM and MTC Reg subsets present in select cases. (f) Dot plot showing the top differentially expressed genes distinguishing each MTC subtype from their benign counterparts (e.g., MTC CM vs. T CM). (g) Venn diagram displaying shared significantly upregulated genes (log_2_ fold change [logFC] > 1, *q* < 0.05) across the three MTC subtypes.

MTCs were identified using integrated TCR clonotypes (*n* = 12), inferred CNVs and transcriptional divergence from HCs. Clusters with >50% of expanded clonotypes were considered malignant, consistent with prior literature (Appendix S1, Figure 3a, Figure S2A).^5^, ^6^ No dominant CDR3 sequences were shared across samples. Ultimately, we identified 34,974 MTCs and 18,094 non‐malignant T cells in CTCL patients.

Unsupervised clustering of T cells from CTCL patients revealed three transcriptionally and clonally distinct malignant subsets: MTC CM (central memory‐like), MTC E/EM (effector/effector memory‐like) and MTC Reg (regulatory/exhausted‐like) (Figure 3b, Appendix S1). CITE‐seq, which measures surface proteins alongside RNA, validated transcriptomic annotations at the protein level. This confirmed increased CCR4 and reduced CD5 and CD7 expression in MTCs. CD25 expression was restricted to MTC Reg, while PD‐1 was observed on a minority of MTC CM cells (Figure S2B). Partition‐based graph abstraction (PAGA) trajectory inference suggested that MTC CM forms a distinct and potentially terminal state, whereas MTC E/EM and MTC Reg exhibited closer connectivity and possible lineage plasticity (Figure 3c).

CNV profiling revealed distinct patterns across malignant subsets: MTC CM and MTC Reg showed recurrent alterations on chromosomes 7, 8 and 17, while MTC E/EM displayed modest, diffuse CNVs across chromosomes 8, 12, 14 and 17 (Figure 3d). Subtype distributions varied by patient: MTC CM comprised 91.7% of all MTCs, compared to smaller MTC E/EM (5.6%) and MTC Reg (2.7%) populations (Figure 3e). Samples with higher circulating tumour burden at time of collection (e.g., ≥60% aberrant CD4^+^ T cells) tended to show larger proportions of these minor malignant subsets (e.g. Pt_10, Pt_14, Pt_19–22 in Pool_2), although these trends were modest and non‐significant.

### Functional signatures of malignant T cells provide insight into biologic activity

To define malignant programmes beyond baseline cell identity, we compared each malignant subtype to its closest benign counterpart (MTC CM vs. T CM, MTC E/EM vs. T EM and MTC Reg vs. T Reg) based on differentially expressed genes (DEG) (Figure 3f, Figure S2D, Tables S5 and S6).

MTC CM exhibited a Th2/Th22‐skewed, immune‐evasive profile with upregulation of genes previously associated with CTCL including *AIRE, CXCL13, TCF7, KIR3DL2* and *NEDD4L*, alongside decreased *CD7*.^6^, ^8^, ^13^, ^14^, ^15^, ^16^ Newly identified *CRIP1* and *GNG4*, implicated in immune suppression and cancer progression in other malignancies, were also enriched (all *q* < 0.0001).^17^, ^18^ While many of these markers have been individually reported, our analysis highlights their collective relevance within a cohesive malignant CD4^+^ T‐cell phenotype.

MTC E/EM demonstrated a stress‐adaptive, metabolically reprogrammed state marked by *FOSB, EGR1, HSPA1B, NR4A1* and *LMNA*.^19^, ^20^, ^21^, ^22^ MTC Reg overlapped with both other subsets, expressing shared immune‐evasive and stem‐like transcripts (e.g., *KIR3DL2, NEDD4L, CD70*) and survival‐associated genes enriched in MTC E/EM (e.g., *LMNA, DUSP4*).^13^, ^23^, ^24^, ^25^ Interleukin (IL)‐2 receptor subunits showed variable expression across malignant‐benign pairs: modest *IL2RA* enrichment only in MTC Reg, *IL2RB* and IL2RG in MTC CM and reduced *IL2RB MTC/*EM, consistent with the reported heterogeneity of CD25 expression in SS.^6^

Despite transcriptional diversity, a conserved 13‐gene signature was upregulated across all malignant subsets. This profile includes *PLS3*, *MLF1*, *GEM*, *DNMO3S*, *HACD1* and *NR4A3*, reflecting common programmes in immunomodulation, proliferation, invasion and metabolic adaptation (Figure 3g).^26^, ^27^, ^28^, ^29^, ^30^, ^31^, ^32^, ^33^, ^34^, ^35^, ^36^

Clinically, MTC E/EM cells were more common in untreated patients (e.g., Pt_10–15), while MTC Reg cells were enriched in those receiving HDAC inhibitors (HDACis) (e.g., Pt_2, Pt_7, Pt_8), consistent with prior associations between *FOXP3* expression and HDACi exposure.^5^ However, these trends were variable and limited by pooled samples and incomplete treatment annotation (Figure 3e, Table 1).

### Altered non‐malignant cell composition and gene expression in CTCL

**FIGURE 4:**
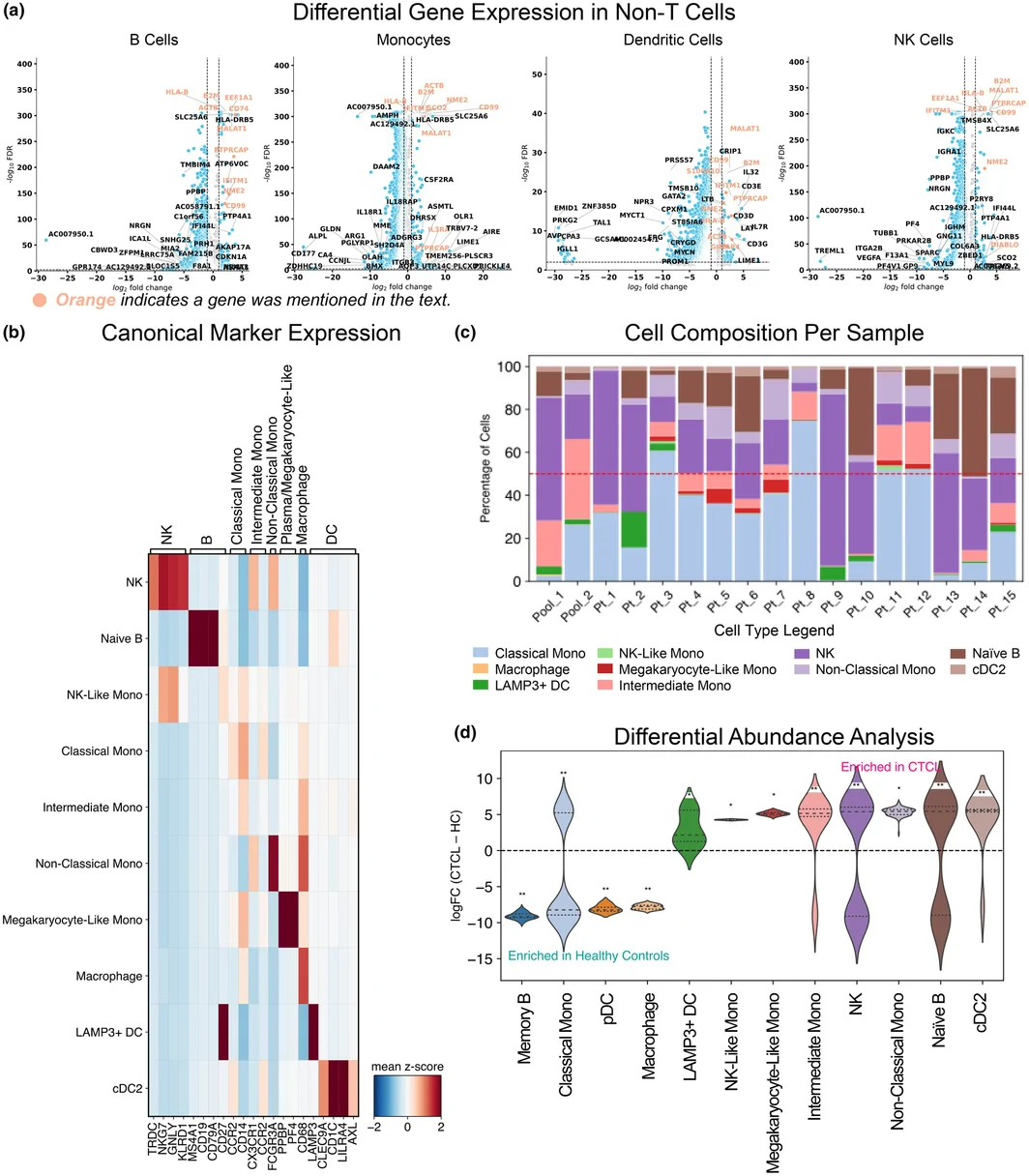
Non‐T cells demonstrate distinct profiles in patients with cutaneous T‐cell lymphoma (CTCL) compared to healthy controls (HCs). (a) Volcano plots showing differentially expressed genes (DEGs) between CTCL patients and HCs in NK cells, DCs, monocytes (Mono) and B cells. Axes represent log2 fold change and –log10 false discovery rate (FDR). (b) Heatmap displaying canonical marker expression across non‐T cell subsets within CTCL patient samples. (c) Bar plot depicting the proportional abundance of non‐T cell populations per sample. (d) Violin plots comparing cell neighbourhood enrichment scores between patients with CTCL and HCs, indicating microenvironmental alterations in non‐T cell spatial context. Statistical significance is annotated as: *p ≤ 0.01, **p ≤ 0.0001. PDC, plasmacytoid dendritic cell.

We next examined how non‐T‐cell compartments differ between CTCL patients and HCs to elucidate disease‐associated remodelling of the immune landscape. DEG analysis revealed widespread transcriptional alterations across B cells, NK cells, monocytes and DCs in CTCL compared to HCs (Figure 4a). Cell‐type assignments were supported by canonical marker expression (Figure 4b) and validated using CITE‐seq in a subset of samples (*n* = 7; Figure S3A).

We identified 25 genes significantly upregulated across all four non‐T‐cell types, including *MALAT1*, *IL32*, *CD99*, *NME2*, *HLA‐B*, *IFITM1*, *B2M*, *ACTB* and *PTPRCAP*, suggesting convergent activation, stress adaptation and migratory signalling.^37^, ^38^, ^39^, ^40^, ^41^, ^42^, ^43^, ^44^, ^45^, ^46^, ^47^, ^48^, ^49^, ^50^, ^51^, ^52^ Mitochondrial gene upregulation indicated metabolic adaptation (Figure 4a, Figure S3B).

Transcriptomic changes also highlighted cell‐specific remodelling in this dysregulated immune landscape. B cells upregulated *CD74*, involved in antigen presentation and tumour‐supportive signalling, and *EEF1A1*, linked to protein synthesis and cellular stress responses (Table S8).^53^, ^54^ Monocytes upregulated *SCO2*, which regulates metabolic responses to stress, and *IL3RA*, indicating chemokine‐driven recruitment and immune modulation (Table S9).^41^, ^55^, ^56^ DCs upregulated *S100A10* and *GIMAP4*, suggesting roles in immune regulation, infiltration and survival (Table S10)^57^, ^58^ NK cells upregulated *EEF1A1*, shared with monocytes and B cells, and *DIABLO*, a pro‐apoptotic gene (Figure 4a, Table S11).^59^

Although cell‐type frequencies varied across individual samples, as previously reported in CTCL, proportional analysis adjusted for inter‐sample variation revealed significant and consistent differences (Figure 4c).^5^, ^7^, ^16^ CTCL samples showed expansion of non‐classical and intermediate monocytes, LAMP3+ DCs and naïve B cells, while classical monocytes, plasmacytoid DCs and mature B cells were more abundant in HCs (Figure 4d, Figure S3C,D). These shifts suggest a reorganization of the non‐T‐cell compartment, likely driven by chronic inflammation and tumour‐mediated reprogramming. Clinical correlations between malignant subtypes and non‐T‐cell populations were limited: samples with more MTC E/EM cells showed small increases in B cells, while those with more MTC Reg cells showed weak associations with intermediate monocytes (Figures 3e and 4d). These patterns were inconsistent across patients and not statistically meaningful.

### Malignant T cells exploit KIR‐family receptors to evade immune surveillance

To contextualize immune interactions, we examined cell–cell communications signalling among MTCs and immune populations. Sézary cells emerged as central hubs with extensive contacts involving NK cells, monocytes, CD8+ T cells and B cells (Figure S4A). This is notable because Sézary cells may either be suppressed by surrounding immune cells or co‐opt inhibitory signalling pathways to promote their own survival or self‐renewal.

Ligand‐receptor analysis revealed heightened signalling through *KIR3DL2*, *KIR3DL1* and *KIR2DL3*, prompting focused evaluation of the KIR axis.^13^, ^60^ While *KIR3DL2* (CD158k) is a well‐established surface marker and investigational target in CTCL, our study is the first, to our knowledge, to report upregulation of *KIR3DL1* and *KIR2DL3* on MTCs in SS.^13^, ^61^

**FIGURE 5:**
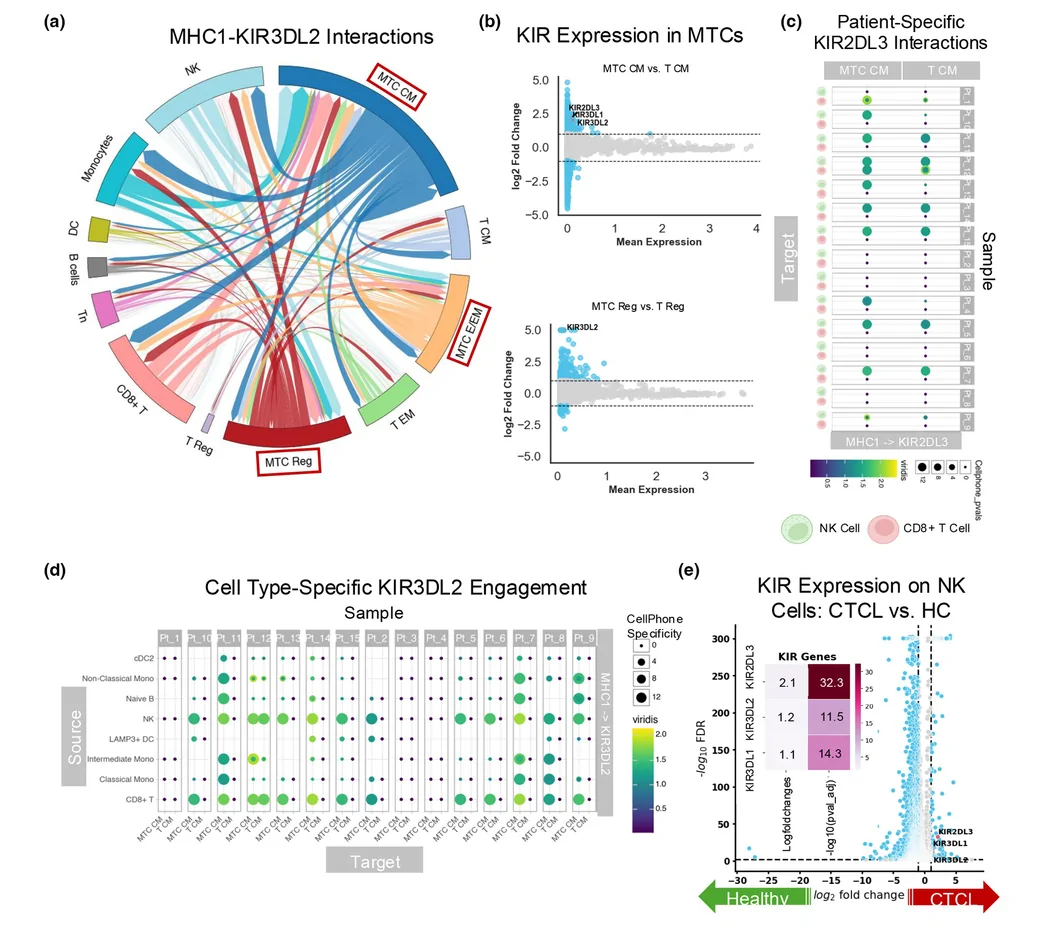
Malignant T CM and T Reg cells engage in bidirectional immunosuppressive interactions via KIR‐family receptors. (a) Circos plot showing increased major histocompatibility complex class I (MHCI)–killer cell immunoglobulin‐like receptor (KIR) 3DL2 interactions between MTC CM and MTC Reg and surrounding immune cell populations. (b) Mean average (MA) plot comparing expression of KIR‐family receptors in MTC CM versus benign T CM, highlighting significant upregulation in malignant cells. (c) Dot plot illustrating patient‐specific signalling from MTC CM to NK and cytotoxic T cells (CD8+ T) via KIR2DL3. (d) Dot plot showing enhanced MHC class I–KIR3DL2 engagement in MTC CM compared to benign T CM, reinforcing its role as a potential therapeutic target. (e) Volcano plot and heatmap showing increased expression of KIR2DL3, KIR3DL1 and KIR3DL2 in NK cells from CTCL patients.

The most significantly elevated interaction was Major Histocompatibility Complex I (*MHCI*) (combined *HLA‐A, HLA‐B, HLA‐C and B2M*)–*KIR3DL2* between MTCs and surrounding cells, especially in MTC CM and MTC Reg (Figure 5a, Figure S4B). Elevated signalling was also observed for *KIR3DL1* and *KIR2DL3*, which extended beyond traditional NK cells to include CD8^+^ T cells, monocytes and B cells (Figure S4C,D). These findings were consistent with DEG analysis, which revealed significant upregulation of *KIR3DL2*, *KIR2DL3* and *KIR3DL1* in MTC CM versus T CM and of *KIR3DL2* in MTC Reg versus T Reg (Figure 5b).

To better understand these interactions at the patient level, we examined source–target cell‐type signalling patterns. In most patients, MTC CM demonstrated stronger outgoing *KIR2DL3*‐mediated interactions with NK and CD8+ T cells compared to benign T CM (Figure 5c). Additionally, across patients, MTCs received more frequent and stronger *KIR3DL2* input from diverse immune cell types including NK cells, CD8^+^ T cells and monocytes compared to their benign counterparts (Figure 5d), supporting a model of widespread bidirectional immune suppression. This pattern of enhanced KIR signalling was further supported by analysis of the broader immune environment, where all three KIR‐family genes of interest were significantly upregulated in NK cells from CTCL versus HCs (Figure 5e). Together, these findings expand the therapeutic relevance of the KIR axis in SS.

### Myeloid JAK/STAT signalling suggests IL‐10/STAT3‐mediated support of malignant T cells

**FIGURE 6:**
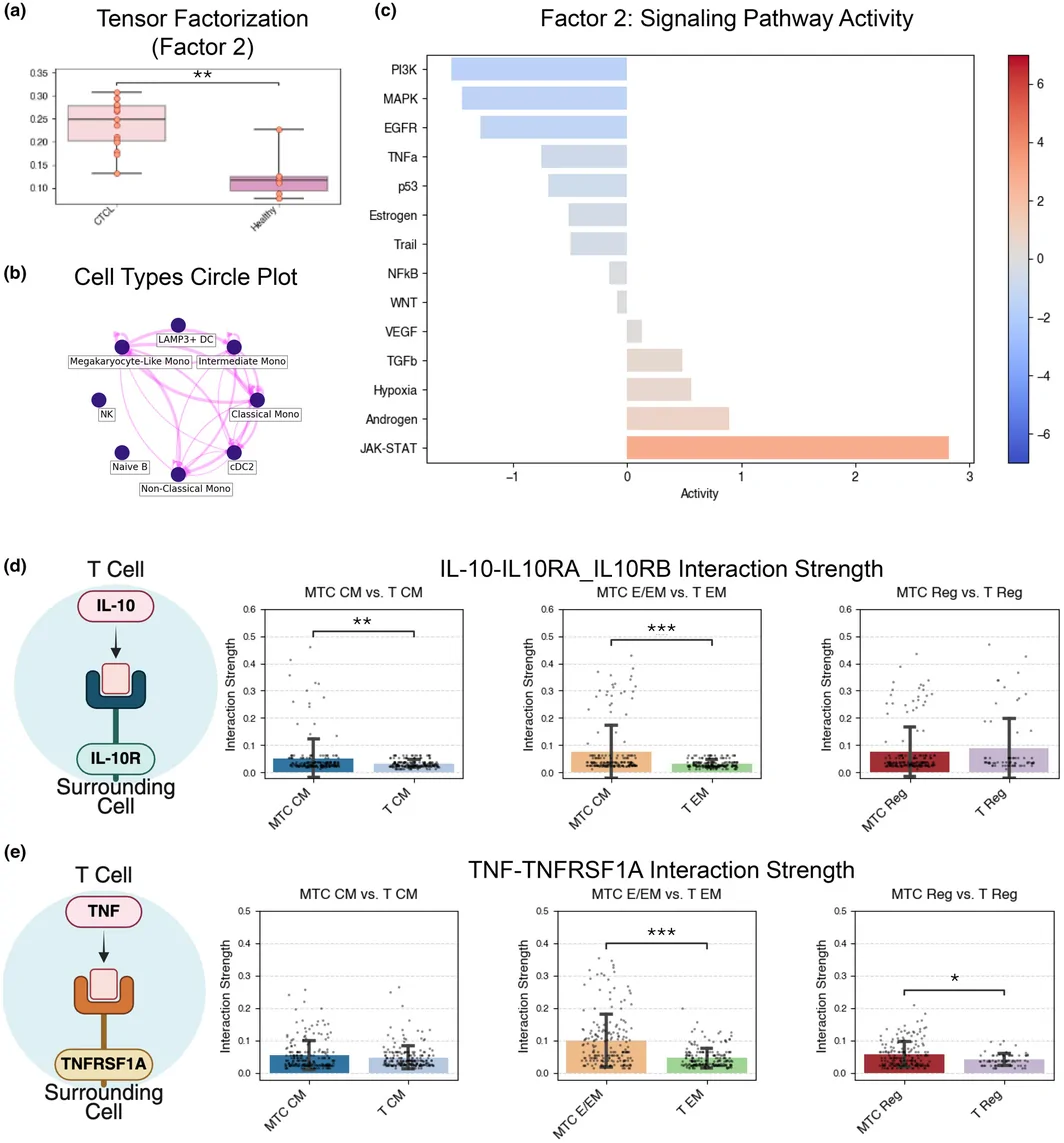
JAK/STAT activation in surrounding myeloid cells suggests targetable signalling pathways. (a) Boxplot showing Factor 2 scores from tensor decomposition analysis (LIANA), demonstrating significantly higher signalling in CTCL patients compared to healthy controls (*q* < 0.01). Each dot represents an individual patient sample. Statistical significance is annotated as: *p ≤ 0.05, **p ≤ 0.01, ***p ≤ 0.001, ****p ≤ 0.0001. (b) Circle plot highlighting the primary cell types contributing to Factor 2, notably monocytes (monos) and dendritic cells (DCs). (c) Horizontal bar plot displaying elevated Janus kinase/signal transducer and activator of transcription (JAK–STAT) pathway activity discovered from Factor 2 in Monos and DCs from CTCL patients relative to healthy controls. (d) Boxplots illustrating significantly stronger interleukin (IL)‐10 → IL10RA_IL10RB interactions from MTCs (MTC CM and MTC E/EM) compared to benign subsets. (e) Boxplots demonstrating increased tumour necrosis factor (TNF) → TNF receptor superfamily member 1A (TNFRSF1A) signalling from MTC E/EM and MTC Reg to other immune cells, suggesting paracrine immunosuppressive effects. Statistical significance is annotated as: *p ≤ 0.01, **p ≤ 0.001, ***p ≤ 0.0001.

To investigate how the microenvironment contributes to immune dysfunction, we modelled ligand‐receptor interactions among non‐T cells from CTCL and HCs. We used tensor factorization, a method that identifies coordinated patterns in complex datasets, to group these interactions into dominant communication programmes.^62^ This analysis uncovered several dysregulated signalling programmes in CTCL, with two (Factors 2 and 5) showing the clearest differences from HCs (Figure 6a,b, Figure S5A,B).

Factor 2, strongly enriched in CTCL (q < 0.0001), captured a dominant signalling module involving *S100A8* and *S100A9* interactions with *CD68, CD36* and *ITGB2* on myeloid cells (Figure S5C). These calgranulin‐mediated interactions, associated with chronic inflammation and myeloid activation, were predominantly active in monocytes and DCs, which exhibited nearly threefold higher JAK/STAT pathway activity versus HCs (Figure 6c). This transcriptional programme aligns with a tumour‐supportive myeloid phenotype observed in other cancers and may contribute to immune suppression via STAT3 activation.^63^, ^64^, ^65^

Supporting this, MTCs exhibited increased *IL‐10–IL10RA_IL10RB* signalling to surrounding immune cells (Figure 6d). As IL‐10 is a potent activator of JAK1/tyrosine kinase 2 (TYK2)–STAT3, this may represent a mechanism by which MTCs reprogramme the microenvironment to favour immune tolerance and tumour persistence.^66^ Additionally, *TNF–TNFRSF1A* interactions were elevated in MTC E/EM and MTC Reg subtypes (Figure 6e), further implicating inflammatory tumour necrosis factor (TNF) signalling in STAT3‐mediated dysregulation.^67^ While this immunoregulatory axis could contribute to tumour persistence, its precise role in CTCL pathogenesis remains unclear, warranting further investigation.

By contrast, Factor 5, enriched in HCs (*q* < 0.0001), reflected homeostatic interactions involving G protein‐coupled receptor (GPCR) signalling (*GNAI2–S1PR4, GNAI2–ADCY7*), chemotaxis (*HMGB1–CXCR4*) and cell adhesion (*ADAM10–CD44*). Reduced activity of *GNAI2*, an immune modulator, may contribute to disrupted cell positioning and uncontrolled T‐cell activation in CTCL.^68^, ^69^

Factors 4 and 6, also enriched in CTCL, featured CD74‐centred immunoregulation (e.g., *MIF–CD74–CXCR4, COPA–CD74* and *APP–CD74*), known to promote STAT3 activation and immune suppression.^70^ Factor 6 additionally featured *LILRB1–MHCI* interactions, suggesting checkpoint‐like inhibition. Broad *CD74* upregulation across non‐T cells in CTCL (log_2_FC = 1.44, *q* < 0.0001) position it as a central node of immune dysfunction and a promising therapeutic target.^71^, ^72^ Lastly, Factor 3 showed elevated *MHC–KIR* signalling among non‐T cells in CTCL, echoing KIR‐driven inhibitory signalling observed in MTCs, though this trend did not reach statistical significance (Figure S5C).

## DISCUSSION

SS is increasingly recognized as a clinically and transcriptionally heterogeneous disease, contributing to its morbidity and mortality.^73^ Here, we present an integrated single‐cell analysis of peripheral blood in SS using scRNA‐seq, CITE‐seq and TCR‐seq to refine the characterization of MTCs, elucidate their interactions with the immune microenvironment and highlight potential therapeutic targets (Figure 1). Prior work identified functionally diverse malignant SS cells.^74^ Here, we systematically delineate three transcriptionally distinct MTC subtypes: MTC CM (central memory‐like, immune evasive, most prominent), MTC E/EM (effector/effector memory‐like, metabolically adapted) and MTC Reg (regulatory/exhausted‐like, immunosuppressive). A conserved signature across subtypes, including *DNM3oS* and *PLS3*, reinforces their potential role in CTCL pathogenesis.^75^ This framework clarifies key biological programmes that shape disease behaviour in SS and highlights immune interactions and signalling pathways that may ultimately support more personalized therapeutic strategies in a disease where treatment remains largely nonspecific.

Each MTC subtype reveals distinct therapeutic vulnerabilities. MTC CM, the most prevalent subtype, displayed Th2/Th22 polarization and central memory markers (*CCR7, SELL, TCF7*), validating that central memory T cells are the archetypal Sézary cell.^76^ It also expressed KIR‐family receptors and *CXCL13*. Lacutamab (anti‐*KIR3DL2*) and *CXCL13‐*targeted therapies (under investigation for inflammatory bowel disease and rheumatoid arthritis) represent promising therapeutic avenues.^77^, ^78^ MTC E/EM showed NR4A family activation, suggesting susceptibility to NR4A‐modulating agents such as GLP‐1R agonists.^79^ MTC Reg expressed *ITGB1*, an investigational target in other cancers and was modestly enriched in HDAC inhibitor‐treated patients.^5^, ^80^ *EPCAM*, broadly upregulated across MTCs, may be targetable via emerging EpCAM‐directed CAR‐T strategies.^81^ These subtype‐specific features align with recent observations that therapeutic exposure and patient‐level immune variation can shift malignant and bystander cell states, reinforcing the importance of an integrated and personalized framework for CTCL management.^16^

Transcriptional remodelling of non‐T cell compartments may support Sézary cell adaptation and persistence, including upregulation of *MALAT1*, a STAT3‐induced transcript that promotes IL‐10 secretion and pro‐tumour myeloid recruitment.^82^, ^83^, ^84^, ^85^ IL‐32, a cytokine implicated in several inflammatory and malignant settings, was also elevated in myeloid cells.^86^ These findings suggest that non‐T cells may contribute to CTCL pathogenesis and represent underexplored therapeutic targets. Our findings contribute early biological insight that, with further validation, may ultimately complement guideline‐driven efforts to refine patient stratification and optimize the timing of curative interventions in CTCL.^87^

Our analysis broadens the role of KIR‐family receptors beyond *KIR3DL2* in SS. *KIR3DL2* is the basis for lacutamab, an emerging therapy with durable responses and improved quality of life in relapsed/refractory SS.^61^ In addition to *KIR3DL2*, we observed upregulation of *KIR2DL3* and *KIR3DL1* across MTCs and innate immune cells, suggesting a self‐reinforcing inhibitory network.^13^, ^61^, ^88^, ^89^, ^90^ While *KIR2DL3* expression has been previously noted in a minor MF subclone, our findings demonstrate broader and more consistent expression across Sézary cells, supporting its relevance as an immune checkpoint.^91^ These findings extend known KIR biology in SS and suggest new immunologic checkpoints that could inform future interventions.

While JAK/STAT signalling has been studied in malignant SS cells, our analysis reveals a novel role for this pathway in surrounding cells. Tensor factorization revealed *S100A8, S100A9* and *CD74*‐mediated signalling converging on STAT3 activation in myeloid cells, partly driven by MTC‐derived *IL‐10* and *TNF–TNFRSF1A*, suggesting that MTCs may reprogramme the surrounding environment to sustain immunosuppression. *S100A9–TLR4* signalling, linked to NF‐κB–driven tumour growth in CTCL, may be reversible with tasquinimod.^92^, ^93^ *CD74*, upregulated across non‐T cells, supported CTCL‐enriched *MIF–CD74* and *CD74–CXCR4* interactions, known STAT3 activators and targetable with agents like STRO‐001.^70^, ^94^ Prior studies reported success with JAK inhibitors in CTCL, although with limited efficacy in MF/SS and potential relapse in immunosuppressed patients.^95^ While our findings suggest that STAT3 activity in myeloid cells may sustain the immunosuppressive niche in CTCL, its functional role in SS remains unclear, warranting further validation to determine whether biomarker‐driven or combination strategies would aid therapy or compromise immune surveillance.

Despite the application of multimodal single‐cell technologies, our modest cohort size (*n* = 22 patients with CTCL) limited detection of treatment‐ or outcome‐specific associations, and sample pooling constrained clinical annotation for a subset of patients. Although TCR sequencing was not available for all samples, malignant identity was robustly assigned using a multiparametric framework incorporating transcriptional divergence, inferred CNVs and canonical markers. Because plasma and fresh cells were not consistently available, we did not perform functional assays; instead, we validated signals by demonstrating convergence across patients using complementary in silico analyses and sensitivity checks. The absence of matched skin samples restricted assessment of circulating–tissue correlations. Delineating potential transitions between MTC subsets will require paired whole‐exome sequencing and/or longitudinal pre‐/post‐treatment sampling, which cannot be resolved within a cross‐sectional design. Future studies with spatial and longitudinal sampling, paired blood–skin profiling and functional validation will be critical to validating malignant subtype dynamics and advancing clinical translation. While the integration of public and prospectively collected datasets enhanced the scope of our analysis, validation in more diverse populations and disease stages is needed to ensure generalizability.

In summary, we identify three malignant T‐cell subtypes in SS—MTC CM, MTC E/EM and MTC Reg—each with distinct transcriptional features and potential therapeutic relevance. Our findings show that immune dysregulation extends beyond malignant cells, involving broader crosstalk with the surrounding immune environment. For clinicians, these data support a more integrated view of SS biology and suggest that effective therapy may ultimately need to address both malignant cells and the immunologic milieu they remodel. These insights offer a biological framework that may inform future decisions regarding targeted therapy selection and treatment sequencing to improve outcomes in CTCL.

## AUTHOR CONTRIBUTIONS

Beth A. Childs led data analysis and investigation, performed formal analysis and visualization and wrote the original draft; Eslam A. Elghonaimy co‐led project conceptualization and methodology, contributed to analysis, validation and visualization, and assisted in both the original draft and reviewing and editing the manuscript; Praveen Ramakrishnan Geethakumari assisted with data curation, investigation and manuscript review; Kiran A. Kumar contributed to project conceptualization, data curation and manuscript review; Joseph F. Merola provided clinical insight, resources and manuscript feedback; Heather W. Goff Goff co‐led project conceptualization, provided funding and supervision and contributed to analysis and manuscript review; Todd A. Aguilera co‐led project conceptualization and methodology, secured funding and resources, oversaw supervision and project administration and contributed to visualization, analysis and manuscript preparation and review.

## FUNDING INFORMATION

This study was supported by the Josephine Hughes Sterling Foundation through a research grant to the UT Southwestern Cutaneous Lymphoma Research Program (HWG).

## CONFLICT OF INTEREST STATEMENT

Praveen Ramakrishnan Geethakumari has provided consultancy services to Kite Pharma, Bristol–Myers Squibb and served on the advisory boards of Pharmacyclics LLC, ADC Therapeutics, Cellectar Biosciences, IPSEN, Acrotech Biopharma and Ono Pharma; Joseph F. Merola serves on the medical and scientific board of the Lupus Foundation of America and is a consultant and/or investigator for Amgen, Astra Zeneca, Boehringer Inhelheim, Bristol–Myers Squibb, Abbvie, Dermavant, Eli Lilly, Incyte, Novartis, Janssen, UCB, Sanofi‐Regeneron, Sun Pharma, Biogen, Pfizer and Leo Pharma; Todd A. Aguilera reports grants from UT Southwestern, CPRIT and the Carroll Shelby Family Foundation during the conduct of the study, as well as grants from the NIH/NCI, the American Cancer Society Brightedge, UT Southwestern and the Damon Runyon Cancer Research Foundation and other interest or support from Galera Therapeutics, Apexigen, Avelas Biosciences, ALPA Biosciences and Canopy Cancer Collective/1440 Foundation and grants from outside the submitted work; in addition, has a patent for US11246940B2 issued, licensed to Avelas Biosciences and royalties paid from UC San Diego, a patent for issued, licensed to AKSO Biosciences and royalties paid from Stanford, and a patent for US.P1/1001319964 pending. Eslam A. Elghonaimy has other support or interest in ALPA Biosciences outside the submitted work, Beth A. Childs, Kiran A. Kumar and Heather W. Goff have no conflicts of interest to disclose.

## ETHICAL APPROVAL

All human sample collection protocols were reviewed and approved by the UT Southwestern Institutional Review Board (IRB approval #STU 052018‐005).

## ETHICS STATEMENT

Written informed consent was obtained from all participants prior to sample collection in accordance with the Declaration of Helsinki and institutional guidelines. The patients in this manuscript have given written informed consent to publication of their case details.

## Supporting information


Appendix S1

Data S1

## Data Availability

Public datasets analysed in this study are available in the Gene Expression Omnibus (GEO) under accession numbers GSE124899, GSE146586, GSE165623 and GSE171811. Processed single‐cell data from UTSW samples will be deposited to GEO upon publication.
