# Supplementary material for: Mapping malignant T‐cell states and immune circuits in Sézary syndrome by single‐cell analysis
Source: J Eur Acad Dermatol Venereol. 2026 Mar 3;40(9):1535–49. doi: 10.1111/jdv.70368 (PMC13505662; doi:10.1111/jdv.70368)
Supplement: Supplementary file 1 [file JDV-40-1535-s001.docx]

**Supplemental Methods**

**Study Design and Setting**

This was a cross-sectional observational study analyzing peripheral blood mononuclear cells (PBMCs) from patients with cutaneous T-cell lymphoma (CTCL), conducted through integration of newly collected and publicly available single-cell RNA-sequencing (scRNA-seq) datasets. Eight newly recruited samples were collected at UT Southwestern (Dallas, Texas, USA) in 2019 under IRB approval (STU 052018-005) with written informed consent, while public data were drawn from four previously published CTCL datasets (GSE124899, GSE146586, GSE165623, GSE171811) and healthy controls (HCs) from GSE171811 and Tabula Sapiens. The study leveraged both retrospective and prospectively collected samples.

**Participants**
PBMCs were collected from 22 CTCL patients: 12 with Sézary syndrome (SS), 2 with leukemic mycosis fungoides (MF), and 8 newly recruited SS patients at UTSW. Inclusion criteria included expert clinical diagnosis of SS and confirmation by flow cytometry, with an average Sézary cell burden of 66% of CD4+ T cells. All UTSW patients provided written informed consent. Seven healthy controls (HCs) were included from public datasets to serve as comparator samples (age range: mean 54 years; 3 females, 4 males). Matching criteria were not applicable as this was not a matched case-control study. Demographic and clinical information is provided in Table 1.

**Variables and Data Sources**

Primary variables of interest included transcriptional profiles, T-cell receptor (TCR) clonotypes, inferred copy number variations (CNVs), and predicted ligand-receptor interactions. Outcomes included malignant T-cell subtype classification, differential gene expression signatures, and cell-cell communication patterns. Cell identity, malignancy status, and subtypes were defined using transcriptomic, clonal, and CNV data. Full methods for public datasets are described in their respective GEO entries.

**Sample Processing and Single-Cell Sequencing**
PBMCs were isolated using Ficoll density gradient centrifugation and processed using 10x Genomics 5′ Single Cell Library & Gel Bead Kit v1.1 or v2. Seven UTSW samples were also analyzed by CITE-seq and flow cytometry for protein validation. TCR sequencing was available for 15 public samples, with methods described in their respective GEO entries.

**Preprocessing and Quality Control**
Raw sequencing data were processed with Cell Ranger v5.0.1 and aligned to the GRCh38 genome. TCR sequences were aligned using the vdj_GRCh38_alts_ensembl-5.0.0 reference. Downstream analyses were performed in Python using AnnData v0.10.3, Scanpy v1.9.6, and scvi-tools v1.0.2.

Quality control (QC) was performed on a per-sample basis using sc.pp.calculate_qc_metrics. Cells were retained if they met the following criteria:

- 200 detected genes
- <25% mitochondrial gene content
- log₁p(UMI/gene counts) and top 20 gene percentages within 5 SD of the median
- Ribosomal content within 3 SD
- Post-filter mitochondrial content <4 SD

Doublets were identified and excluded using an scVI-based model. A small number of CD4⁺/CD8⁺ double-positive T cells were also removed.

**Clonotype and Malignancy Assignment**
Clonotypes were defined by shared CDR3 amino acid sequences. Expanded clonotypes (top 11 per sample) were labeled C1–C11. In samples with TCR data (n=12), malignant identity was assigned when a single clonotype comprised >50% of a given T cell cluster. In samples without TCR-seq, malignancy was inferred through a multiparameter approach including:

1. Transcriptional divergence from healthy controls
2. Inferred CNVs (via infercnvpy v0.5.1.dev1+ge5943ea)
3. Canonical markers (e.g., decreased CD7, increased AIRE, KIR3DL2 expression)

This framework was benchmarked in TCR-annotated samples for consistency.

**Subtype and Clonality Analysis**
Three malignant T cell (MTC) subtypes were annotated based on canonical markers:

- MTC CM: *CCR7, SELL, TCF7*
- MTC E/EM: *CCR4, CD69, GADD45B*
- MTC Reg: *FOXP3, CTLA4, IL12RA*

Within-patient clonotype analysis showed most malignant cells belonged to a dominant clone. Subtype-specific clonotype enrichment was not consistently observed, supporting phenotypic heterogeneity within individual clones.

**Study Size and Statistical Reporting**

**No formal power calculation was conducted due to the exploratory nature of the study. Sample size was based on data availability, balancing phenotypic and technical diversity. Follow-up duration was not applicable, as this was a cross-sectional study. For all DEG and cell communication analyses, Benjamini-Hochberg FDR correction was applied (q < 0.05).**

**Batch Correction and Integration**
To integrate diverse datasets and mitigate batch effects, we benchmarked multiple approaches and selected Harmony for optimal alignment. Highly variable genes were selected after filtering out ribosomal and mitochondrial transcripts. Batch mixing was assessed by visual inspection of UMAPs and sample contributions across clusters.

**Clustering and Annotation**
Dimensionality reduction was performed using PCA followed by neighbor graph construction (n_neighbors=10, n_pcs=30), Leiden clustering, and UMAP visualization. Cell types were annotated using CellTypist v1.6.3 with reference atlases and manual refinement.

**CNV Inference**
Infercnvpy was used to infer CNVs within T cell clusters, using healthy T cells as a reference. CNV scores were computed per cell and visualized at both cluster and patient levels for validation.

**Differentially Expressed Genes (DEG)**
We employed two complementary frameworks, diffxpy (v0.7.4) and scvi-tools (v1.0.2), for DEG analysis to ensure reproducibility and reduce bias. Analyses included:

- Global comparisons of malignant vs. benign T cells
- Pairwise comparisons of MTC subtypes vs. their benign phenotypic analogues
- Differential expression across non–T cell populations (monocytes, dendritic cells, NK cells, B cells)

Only genes with adjusted q <0.05 and absolute log₂FC >0.5 were considered significant. Lowly expressed genes and technical artifacts (e.g., TRBV, MT, RP genes) were excluded. We prioritized genes consistently identified by both methods to ensure robust, generalizable results. Technical artifacts (e.g., TRBV, MT, RP genes) and lowly expressed genes were excluded. Missing gene values (due to dropout) were handled using model-based approaches embedded in the scVI framework, which uses Bayesian inference to account for sparsity. Sensitivity analyses were conducted by verifying consistency of top genes across both methods and comparing DEG with and without pooled samples.

**Cell–Cell Communication Analysis**
LIANA v1.2.1 was used to infer ligand–receptor interactions at the sample level for non-pooled data (n=15 samples), with 10,000 permutations. Significant interactions (q < 0.05) were used to build a 4D communication tensor, which was decomposed using Cell2Cell’s to_tensor_c2c function. PROGENy was used for pathway activity mapping. KIR–MHC-I interactions were interpreted in the context of KIR variability across malignant subtypes, rather than uniform MHC-I expression.

**Sample Pooling and Attribution**
Public datasets contained demultiplexed, patient-specific samples. At UTSW, seven samples were inadvertently pooled into two batches (Pool1 and Pool2) due to hashtag oligonucleotide dropout. These pooled samples were retained for analyses not requiring individual resolution (e.g., cell–cell communication), but excluded from subtype frequency and per-patient heterogeneity analyses. Thus, most patient-specific analyses include 15 individually resolved samples. Efforts to address potential bias from pooling included cross-validation of subtype expression trends and exclusion from patient-level proportions. Additionally, complementary analytical methods were employed to validate findings, with cell–cell communication results contextualized against differential gene expression analyses both across the full cohort and within individual samples to ensure consistency and interpretative robustness.
