# Supplementary material for: Mapping malignant T‐cell states and immune circuits in Sézary syndrome by single‐cell analysis
Source: J Eur Acad Dermatol Venereol. 2026 Mar 3;40(9):1535–49. doi: 10.1111/jdv.70368 (PMC13505662; doi:10.1111/jdv.70368)
Supplement: Supplementary file 2 [file JDV-40-1535-s002.docx]

**Supplementary Figure 1: Quality control and data integration**

(a) Schematic representation of quality control (QC) workflow, including sample-by-sample QC via ridge plots, doublet removal, merging, and integration using Harmony. (b) UMAP embedding post-Harmony integration, colored by patient sample IDs, illustrating effective data integration. (c) UMAP plots of the entire dataset highlighting lineage-specific gene expression scores (NK, B cells, T cells, myeloid cells) and canonical markers CD8A and CD4. (d) UMAP visualization of integrated T cells colored by patient IDs, demonstrating cell distribution across samples. (e) UMAP plots displaying T-cell phenotype-specific expression scores (CM, EM, Th1, Th2, Th22, Tregs, exhaustion). (f) UMAP of integrated non-T cells colored by patient sample IDs, highlighting distribution across samples. (g) UMAP plots demonstrating phenotype-specific expression scores, identifying subsets such as classical, intermediate, non-classical monocytes, dendritic cells, and rare populations.

*Abbreviations: Th, T-helper cell; T Reg, T-regulatory cell; CM, central memory; EM, effector memory; Class Mono, classical monocyte; Int Mono, intermediate monocyte; Non-Class Mono, non-classical monocyte; cDC1, type 1 conventional dendritic cell; cDC2, type 2 conventional dendritic cell; Mature B, mature B cell; Naïve B, naïve B cell.*

**Supplementary Figure 2: Characterization of malignant T cells**

(a) Pie charts displaying clonal distribution within individual patient samples, highlighting up to two dominant T-cell clones per patient. (b) UMAP plots from CITE-seq data validating protein expression of selected markers (CD8, CD4, CD5, CD7, CCR4, CD45RO, PD1, CD25) in comparison to scRNA-seq data (limited to UT Southwestern samples). (c) Volcano plots showing differentially expressed genes in malignant T-cell subtypes versus their benign counterparts (MTC CM vs. T CM, MTC E/EM vs. T EM, MTC Reg vs. T Reg).

*Abbreviations: Th, T-helper cell; T Reg, T-regulatory cell; CM, central memory; EM, effector memory; MTC, malignant T cell; MTC CM, malignant central memory T cell; MTC E/EM, malignant effector/effector memory T cell; MTC Reg, malignant regulatory T cell;* *UMAP, Uniform Manifold Approximation and Projection;* *CITE-seq, Cellular Indexing of Transcriptomes and Epitopes by Sequencing; scRNA-seq, single-cell RNA sequencing.*

**Supplementary Figure 3: Comparative analysis of non-T cells between CTCL and healthy controls**

(a) UMAP feature plots of CITE-seq-derived surface proteins CD11b, CD19, and CD56 projected onto transcriptomic UMAP, validating non-T cell subset definitions. (b) Venn diagram summarizing overlaps in differentially expressed genes across B cells, DCs, monocytes, and NK cells, with highlighted genes significantly upregulated in both groups. (c) UMAP visualization distinguishing non-T cell subsets across healthy control and CTCL samples. (d) UMAP visualization showing clustering of non-T cell populations by disease condition, highlighting distinct clustering of classical monocytes in CTCL.

*Abbreviations: UMAP, Uniform Manifold Approximation and Projection; CITE-seq, Cellular Indexing of Transcriptomes and Epitopes by Sequencing; CTCL, cutaneous T-cell lymphoma; NK, natural killer cells; DC, dendritic cells; Mono, monocytes; cDC2, type 2 conventional dendritic cells; DCs, dendritic cells; Memory B, memory B cells; Naïve B, naïve B cells.*

**Supplementary Figure 4: Expanded analysis of interactions and MHC I-KIR activity in MTCs**

(a) Heatmap of ligand-receptor interaction strength (lr_means), highlighting malignant T cells as primary sources of enhanced MHC class I interactions with KIR-family receptors. (b) Boxplots confirming significantly stronger KIR3DL2 interactions across all malignant subtypes (MTC CM, MTC E/EM, MTC Reg), emphasizing its relevance in immune evasion and therapeutic potential. (c) Boxplots comparing interaction strengths between malignant and benign T-cell subsets, demonstrating significant elevation in MTC CM and MTC Reg. (d) MHC Class I–KIR3DL1 Interactions: Boxplots highlighting significantly increased interactions in MTC CM and MTC Reg versus benign counterparts, noting elevated signaling in benign T EM compared to MTC E/EM.

*Abbreviations: KIR, Killer Cell Immunoglobulin Like Receptor; MTC, malignant T cell; MTC CM, malignant central memory T cell; T CM, benign central memory T cell; MTC E/EM, malignant effector/effector memory T cel; T EM, benign effector memory T cell; MTC Reg, malignant regulatory T cell; T Reg, benign T-regulatory cell; MHC, major histocompatibility complex; KIR, killer immunoglobulin-like receptor.*

**Supplementary Figure 5: Tensor Factorization Identifies Dominant Signaling Patterns**

(a) Tensor factorization results (LIANA) illustrating major communication themes across cell types. (b) Boxplots showing differences in tensor factor expression between CTCL patients and healthy controls. (c) Heatmap detailing the strongest ligand-receptor interactions identified within each tensor factor.

*Abbreviations: CTCL, cutaneous T-cell lymphoma; NK, natural killer cells; DC, dendritic cells; Mono, monocytes; cDC2, type 2 conventional dendritic cells; DCs, dendritic cells; cDC2, type 2 conventional dendritic cells; Naïve B, naïve B cells.*

**SUPPLEMENTARY TABLES**

| **Pt** | **Sample Date** | **Diagnosis & Early Course** | **Therapies Received (up to and after sampling)** | **Outcomes After Sampling (as of December 2025)** |
| --- | --- | --- | --- | --- |
| **1** | 1/30/19 | MF diagnosed 2015 after rash began in 2014; erythroderma; bone marrow with 10% atypical T cells; recurrent LAD by 2018; progressive skin disease | **Pre-sampling:** mechlorethamine gel, ECP (2015-2017 intermittently), romidepsin (2016-2019).  **At sampling:** NBUVB and romidepsin.  **Post-sampling**: mogamulizumab (2019), PET response (2019), TSEBT (2020), brentuximab (2022), mogamulizumab re-trial. | Progressive CTCL despite therapy; transitioned to hospice 2/2023; **died 3/2023**. |
| **16** | 1/29/20 | MF diagnosed 2019 with +TCR; minimal blood involvement; clinically stable | **Pre-sampling**: ECP (2019–2020), IFNa (2019-2020).  **At sampling**: IFNa 3 MU BIW.  **Post-sampling**: continued IFNa (2021-2023), ECP restart (2023–2025), IFN-γ trial (2023-2025), mogamulizumab (3/2025). | **Deep remission**: clear blood flow and clear skin; mogamulizumab tapering to q2 months. Alive and well. |
| **17** | 3/27/19 | Longstanding rash; SS diagnosed 2018 initially with >90% blood involvement and LAD; erythroderma | **Pre-sampling**: Cyclosporine.  **At sampling**: romidepsin.  **Post-sampling:** Extended romidepsin course (2019-2024), ECP, NBUVB, acitretin, mogamulizumab (2024-2025). | **Marked improvement** on mogamulizumab with controlled rash; near-clear skin; awaiting SCT donor. |
| **18** | 6/5/19 | SS diagnosed early 2019; erythroderma; circulating malignant T cells; early systemic treatment | **Pre-sampling**: MMF (2018), adalimumab (2018, suspected psoriasis), ECP (2019), topical TAC, bleach baths.  **At sampling:** IFNa, was planning to re-initiate ECP.  **Post-sampling**: ECP, TSEB, romidepsin (2019–2024), bexarotene, mogamulizumab (4-10/2024), continued high-dose bexarotene. | **Clear blood flow cytometry** and **PET NED** on bexarotene. Stopped mogamulizumab due to rash. Considering allo-SCT. |
| **19** | 5/29/19 | Erythrodermic SS; full staging incomplete; frail, cachectic trajectory emerging | **Pre-sampling**: none (treatment-naïve).  **At sampling**: no systemic therapy initiated; staging planned.  **Post-sampling**: multiple hospitalizations for UTIs, functional decline, hospice enrollment. | **Lost to follow-up** shortly after 2019; survival unknown. |
| **20** | 4/10/19 | Skin with ~60% BSA involvement; wheelchair-bound; significant functional limitations | **Pre-sampling**: started on acitretin 10 mg daily 4/2019, topical triamcinolone; NBUVB was planned before lost to follow up.  **At sampling**: acitretin 10 mg daily, taking inconsistently.  **Post-sampling**: hospice for home-health support; no additional CTCL-directed therapy documented. | **Lost to follow-up** after 2019; survival unknown. |
| **21** | 3/27/19 | MF/SS diagnosed 2017; heavily pre-treated; persistent blood involvement | **Pre-sampling**: IFNa + ECP (2017-2019), romidepsin (2018), acitretin (2017-2018), targeted radiation.  **At sampling**: bexarotene.  **Post-sampling**: mogamulizumab (2022), brentuximab (2022), myeloablative MUD allo-PBSCT (11/2022), treatment for skin GVHD (steroids/tacrolimus/ruxolitinib). | **Sustained complete remission ≥2 years post-allo-SCT**; no active CTCL or GVHD; stable labs. |
| **22** | 3/27/19 | SS diagnosed ~2012 with blood and nodal disease; long history of treatment-dependent control | **Pre-sampling:** IFNa + ECP (2012–2015 remission), retinoids, NBUVB.  **At sampling**: romidepsin.  **Post-sampling**: gemcitabine (4/2019), 2x doses mogamulizumab (6/2019), then doxorubicin very briefly; developed tumor lysis syndrome 8/2019. | **Died 9/2019** of rapidly progressive, treatment-refractory CTCL. |

**Table S1. Clinical course and outcomes for UT Southwestern (UTSW) patients with leukemic CTCL included in this study.**

Clinical characteristics, therapies, and longitudinal outcomes for patients treated at UTSW whose peripheral blood samples were included in the study. Outcomes are shown only for institutional patients (Pt_1, Pt_16-22), as detailed clinical follow-up was not available for publicly sourced datasets. Outcomes reflect the most recent available chart review (as of November 2025). *Abbreviations: MF, mycosis fungoides; SS, Sézary syndrome; BSA, body surface area; LAD, lymphadenopathy; TCR, T-cell receptor; IFNa, interferon-α, IFNγ, interferon-γ; ECP, extracorporeal photopheresis; NBUVB, narrowband ultraviolet B phototherapy; UVA1, ultraviolet A1 phototherapy; TSEBT, total skin electron beam therapy; SCT, stem-cell transplant; MUD, matched unrelated donor; allo-PBSCT, allogeneic peripheral blood stem-cell transplant; GVHD, graft-versus-host disease; PET NED, positron emission tomography with no evidence of disease; PR, partial response; CR, complete remission.*

| **Cluster** | **HC_1** | **HC_2** | **HC_3** | **HC_4** | **HC_5** | **HC_6** | **HC_7** | **Pool_1** | **Pool_2** | **Pt_1** | **Pt_2** | **Pt_3** | **Pt_4** | **Pt_5** | **Pt_6** | **Pt_7** | **Pt_8** | **Pt_9** | **Pt_10** | **Pt_11** | **Pt_12** | **Pt_13** | **Pt_14** | **Pt_15** |
| --- | --- | --- | --- | --- | --- | --- | --- | --- | --- | --- | --- | --- | --- | --- | --- | --- | --- | --- | --- | --- | --- | --- | --- | --- |
| **CD8+ T** | 296 | 509 | 1093 | 0 | 221 | 1 | 396 | 130 | 457 | 337 | 171 | 388 | 715 | 35 | 131 | 129 | 115 | 1071 | 243 | 256 | 55 | 346 | 156 | 318 |
| **Class Mono** | 1348 | 6445 | 3122 | 252 | 1899 | 1384 | 872 | 4 | 255 | 312 | 16 | 1132 | 453 | 132 | 180 | 692 | 1207 | 5 | 52 | 1951 | 294 | 22 | 86 | 106 |
| **Int Mono** | 149 | 212 | 34 | 0 | 22 | 3 | 36 | 34 | 361 | 32 | 0 | 124 | 93 | 29 | 25 | 120 | 208 | 3 | 4 | 614 | 110 | 3 | 53 | 41 |
| **LAMP3+ DC** | 0 | 0 | 0 | 0 | 0 | 0 | 0 | 6 | 24 | 1 | 17 | 61 | 3 | 0 | 1 | 1 | 5 | 55 | 15 | 2 | 1 | 3 | 8 | 15 |
| **MTC CM** | 0 | 0 | 0 | 0 | 0 | 0 | 0 | 152 | 1581 | 72 | 2804 | 20377 | 276 | 101 | 411 | 486 | 645 | 1348 | 887 | 293 | 803 | 298 | 672 | 866 |
| **MTC E/EM** | 0 | 0 | 0 | 0 | 0 | 0 | 0 | 120 | 868 | 3 | 30 | 139 | 11 | 2 | 9 | 8 | 13 | 19 | 439 | 7 | 15 | 15 | 78 | 177 |
| **MTC Reg** | 0 | 0 | 0 | 0 | 0 | 0 | 0 | 18 | 386 | 1 | 192 | 38 | 5 | 6 | 8 | 11 | 16 | 12 | 206 | 6 | 9 | 3 | 15 | 17 |
| **MP** | 3 | 391 | 7 | 0 | 22 | 14 | 0 | 1 | 1 | 0 | 0 | 0 | 0 | 0 | 0 | 0 | 0 | 0 | 0 | 1 | 0 | 0 | 0 | 0 |
| **ML Mono** | 0 | 0 | 0 | 0 | 0 | 0 | 0 | 0 | 0 | 2 | 0 | 43 | 16 | 25 | 13 | 102 | 6 | 0 | 1 | 94 | 13 | 1 | 0 | 5 |
| **Memory B** | 130 | 89 | 171 | 0 | 213 | 2 | 118 | 0 | 0 | 0 | 0 | 0 | 0 | 0 | 0 | 0 | 0 | 0 | 0 | 0 | 0 | 0 | 0 | 0 |
| **NK** | 192 | 462 | 1951 | 0 | 340 | 18 | 290 | 91 | 201 | 611 | 51 | 223 | 284 | 56 | 150 | 361 | 70 | 702 | 241 | 383 | 41 | 418 | 340 | 97 |
| **NK-Like Mono** | 0 | 0 | 0 | 0 | 0 | 0 | 0 | 0 | 0 | 3 | 0 | 22 | 2 | 0 | 1 | 8 | 0 | 0 | 0 | 75 | 0 | 0 | 0 | 0 |
| **Naive B** | 258 | 186 | 390 | 0 | 439 | 0 | 165 | 18 | 32 | 2 | 13 | 39 | 174 | 58 | 150 | 75 | 0 | 80 | 230 | 24 | 44 | 229 | 513 | 120 |
| **NC Mono** | 0 | 0 | 0 | 0 | 0 | 0 | 0 | 1 | 65 | 12 | 3 | 183 | 86 | 54 | 29 | 316 | 108 | 23 | 17 | 555 | 53 | 48 | 9 | 52 |
| **T CM** | 776 | 435 | 249 | 0 | 101 | 2 | 1275 | 11 | 199 | 59 | 3290 | 2378 | 531 | 106 | 161 | 199 | 106 | 1064 | 852 | 84 | 134 | 385 | 184 | 563 |
| **T EM** | 150 | 160 | 49 | 0 | 37 | 0 | 586 | 10 | 79 | 29 | 475 | 535 | 22 | 2 | 7 | 54 | 173 | 28 | 21 | 65 | 45 | 19 | 267 | 72 |
| **T Reg** | 194 | 95 | 135 | 0 | 32 | 8 | 194 | 2 | 6 | 0 | 24 | 1 | 41 | 0 | 1 | 2 | 1 | 13 | 5 | 0 | 0 | 4 | 8 | 4 |
| **Tn** | 43 | 124 | 298 | 0 | 49 | 0 | 39 | 8 | 47 | 1 | 174 | 65 | 27 | 2 | 8 | 10 | 9 | 48 | 137 | 4 | 52 | 23 | 35 | 70 |
| **cDC2** | 0 | 1 | 0 | 0 | 0 | 0 | 76 | 4 | 29 | 6 | 2 | 35 | 19 | 10 | 25 | 26 | 12 | 12 | 3 | 65 | 7 | 24 | 7 | 24 |
| **pDC** | 7 | 21 | 43 | 0 | 27 | 2 | 10 | 0 | 0 | 0 | 0 | 0 | 0 | 0 | 0 | 0 | 0 | 0 | 0 | 0 | 0 | 0 | 0 | 0 |

**Table S2.** **Cell counts per cluster and sample after quality control (QC) filtering**

*CD8+ T: cytotoxic CD8+ T cells, Class Mono: classical monocytes, Int Mono: intermediate monocytes, DC: dendritic cells, MTC: malignant T cells, CM: central memory, E/EM: effector/memory, Reg: regulatory, MP: macrophage, ML Mono: megakaryocyte-like monocytes, B: B cells, NK: natural killer cells, NK-Like Mono: natura killer cell-like monocytes, NC Mono: nonclassical monocytes, Tn: T-naïve cells, cDC2: type 2 conventional dendritic cells, pDC plasmacytoid dendritic cells, cDC2 type 2 conventional dendritic cells*

| **Score Categories** | ***Marker Genes*** |
| --- | --- |
| NK Cell | *NKG7, GNLY, KLRD1, NCAM1, CD244* |
| Myeloid Cell | *CD68, ITGAM, CD24, ADGRE1, ITGAE* |
| B Cell | *MS4A1, CD19, CD79A, CD79B* |
| T Cell | *CD3D, CD3E, CD3G, CD4, CD8A, CD7, CD5* |
| Th1 | *GADD45B, IFNG, STAT4, TNF* |
| Th2 | *CCR6, GATA3* |
| Th22 | *AHR, CCR4, CCR6, CCR10, IL22* |
| T Reg | *FOXP3, IL12RA, CTLA4, TIGIT* |
| T Cell Exhaustion | *CXCL13, TIGIT* |
| T Cell Cytotoxicity | *GZMA, PRF1, CCL3, CCL4, IFNG, GZMB, NKG7, GZMB, GNLY* |
| T Central Memory | *CCR7, SELL, TCF7, PTPRC* |
| T Effector Memory | *CCR4, CD69, ITGAE, PTPRC, FAS* |
| Classical Monocyte | *CCR2, CD14, CCR5* |
| Intermediate Monocyte | *CD68, CCR2, CD14, CCR5, FCGR3A, FCGR3B* |
| Non-Classical Monocyte | *FCGR3A, FCGR3B, CXCR1, CX3CR1* |
| cDC1 | *BTLA, CADM1, ITGAE, ITGAX* |
| cDC2 | *CD14, CD163, CLEC10A, NOTCH2, CX3CR1, CD1C, CD2, ID2, IRF4, KLF4* |
| Mature B Cell | *CD38, CR2, FCER2* |
| Naïve B Cell | *IGHD, CD19* |

**Table S3. Gene sets used for cell type and functional state scoring (related to Supplementary Figure 1C, 1E, and 1G)**

Table S3 lists the gene sets used to calculate cell type–specific and functional state scores shown in Supplementary Figure 1C, 1E, and 1G. Scores were computed by averaging expression of marker genes previously validated in the literature for each category. *Abbreviations: NK, natural killer; Th, T helper; Treg, regulatory T celll; DC, dendritic cell; cDC1/cDC2, conventional dendritic cell type 1/2.*

| **Cluster** | **Gene** | **p_val** | **avg_log2FC** | **p_val_adj** |
| --- | --- | --- | --- | --- |
| CD8+ T | CCL5 | 0 | 4.742620468 | 0 |
| CD8+ T | NKG7 | 0 | 4.555574894 | 0 |
| CD8+ T | GZMA | 0 | 3.732661724 | 0 |
| CD8+ T | CST7 | 0 | 2.960759878 | 0 |
| CD8+ T | GZMH | 0 | 4.707960129 | 0 |
| CD8+ T | CTSW | 0 | 3.122534275 | 0 |
| CD8+ T | PRF1 | 0 | 3.268373489 | 0 |
| CD8+ T | FGFBP2 | 0 | 3.905147076 | 0 |
| CD8+ T | GZMB | 0 | 3.408941984 | 0 |
| CD8+ T | HCST | 0 | 1.606443405 | 0 |
| CD8+ T | GZMM | 0 | 2.790809631 | 0 |
| CD8+ T | GNLY | 0 | 3.311241865 | 0 |
| CD8+ T | CD8A | 0 | 4.790156841 | 0 |
| CD8+ T | CCL4 | 0 | 3.226287365 | 0 |
| CD8+ T | KLRD1 | 0 | 3.234349251 | 0 |
| CD8+ T | CD2 | 0 | 2.02773428 | 0 |
| CD8+ T | HOPX | 0 | 2.516045332 | 0 |
| CD8+ T | CD8B | 0 | 4.306833744 | 0 |
| CD8+ T | C12orf75 | 0 | 2.066260099 | 0 |
| CD8+ T | KLRG1 | 0 | 3.430784225 | 0 |
| CD8+ T | RARRES3 | 0 | 1.571729064 | 0 |
| CD8+ T | LYAR | 0 | 2.305831909 | 0 |
| CD8+ T | SYNE2 | 0 | 1.563319564 | 0 |
| CD8+ T | SYNE1 | 0 | 2.027023554 | 0 |
| CD8+ T | UBB | 0 | 0.984384835 | 0 |
| Class Mono | S100A8 | 0 | 5.319198132 | 0 |
| Class Mono | S100A9 | 0 | 4.986746311 | 0 |
| Class Mono | S100A12 | 0 | 5.884672642 | 0 |
| Class Mono | LYZ | 0 | 5.227620602 | 0 |
| Class Mono | MNDA | 0 | 5.333661556 | 0 |
| Class Mono | FCN1 | 0 | 5.050788879 | 0 |
| Class Mono | CTSS | 0 | 3.418771029 | 0 |
| Class Mono | VCAN | 0 | 5.776087761 | 0 |
| Class Mono | LST1 | 0 | 4.009200096 | 0 |
| Class Mono | MS4A6A | 0 | 5.210998058 | 0 |
| Class Mono | CSTA | 0 | 5.161288261 | 0 |
| Class Mono | AIF1 | 0 | 4.098974228 | 0 |
| Class Mono | GRN | 0 | 3.655825138 | 0 |
| Class Mono | SERPINA1 | 0 | 4.160812378 | 0 |
| Class Mono | CST3 | 0 | 3.873757362 | 0 |
| Class Mono | TYMP | 0 | 3.534389734 | 0 |
| Class Mono | SPI1 | 0 | 4.144917488 | 0 |
| Class Mono | NCF2 | 0 | 4.928340912 | 0 |
| Class Mono | TKT | 0 | 2.821470499 | 0 |
| Class Mono | TALDO1 | 0 | 2.789353371 | 0 |
| Class Mono | CD14 | 0 | 5.298225403 | 0 |
| Class Mono | TYROBP | 0 | 3.517752647 | 0 |
| Class Mono | PSAP | 0 | 2.625902176 | 0 |
| Class Mono | CEBPD | 0 | 3.806642294 | 0 |
| Class Mono | APLP2 | 0 | 3.678227186 | 0 |
| Int Mono | CST3 | 0 | 3.925885439 | 0 |
| Int Mono | SAT1 | 0 | 2.802797794 | 0 |
| Int Mono | HLA-DRA | 0 | 3.576267958 | 0 |
| Int Mono | PSAP | 0 | 2.739881516 | 0 |
| Int Mono | TYROBP | 0 | 3.314064503 | 0 |
| Int Mono | AIF1 | 0 | 3.19081378 | 0 |
| Int Mono | FCER1G | 0 | 2.936057806 | 0 |
| Int Mono | HLA-DRB1 | 0 | 3.432316065 | 0 |
| Int Mono | FCN1 | 0 | 3.259858608 | 0 |
| Int Mono | LYZ | 0 | 3.276810408 | 0 |
| Int Mono | SERPINA1 | 0 | 3.029562235 | 0 |
| Int Mono | CTSS | 0 | 2.593741894 | 0 |
| Int Mono | SPI1 | 0 | 2.725101948 | 0 |
| Int Mono | LST1 | 0 | 2.776778221 | 0 |
| Int Mono | NEAT1 | 0 | 2.320338726 | 0 |
| Int Mono | TYMP | 0 | 2.538033962 | 0 |
| Int Mono | CD74 | 0 | 3.025821209 | 0 |
| Int Mono | HLA-DPA1 | 0 | 2.850233793 | 0 |
| Int Mono | S100A8 | 0 | 3.552746058 | 0 |
| Int Mono | FTL | 0 | 2.686908007 | 0 |
| Int Mono | CFD | 0 | 2.870903492 | 0 |
| Int Mono | FTH1 | 0 | 2.44031477 | 0 |
| Int Mono | S100A9 | 0 | 3.122757912 | 0 |
| Int Mono | HLA-DPB1 | 0 | 2.721873999 | 0 |
| Int Mono | NPC2 | 0 | 2.133535385 | 0 |
| LAMP3+ DC | MALAT1 | 1.32E-22 | 1.27797246 | 2.971E-18 |
| LAMP3+ DC | IL32 | 3.92E-22 | 1.396389842 | 4.396E-18 |
| LAMP3+ DC | RPS26 | 1.07E-16 | 0.794490278 | 5.975E-13 |
| LAMP3+ DC | MTRNR2L12 | 3.84E-15 | 1.907307863 | 1.229E-11 |
| LAMP3+ DC | B2M | 8.25E-15 | 0.891773283 | 2.124E-11 |
| LAMP3+ DC | TMSB10 | 1.12E-14 | 0.771938741 | 2.512E-11 |
| LAMP3+ DC | CD52 | 1.78E-14 | 0.914542675 | 3.621E-11 |
| LAMP3+ DC | VIM | 1.29E-13 | 0.679937482 | 1.7E-10 |
| LAMP3+ DC | CD3E | 2.68E-13 | 0.921808064 | 2.868E-10 |
| LAMP3+ DC | RPS21 | 4.84E-13 | 0.516103804 | 4.522E-10 |
| LAMP3+ DC | RPL41 | 4.02E-12 | 0.672854125 | 2.917E-09 |
| LAMP3+ DC | RPS12 | 4.14E-12 | 0.661908209 | 2.917E-09 |
| LAMP3+ DC | POTEE | 5.71E-12 | 7.720767021 | 3.746E-09 |
| LAMP3+ DC | IFITM1 | 8.52E-12 | 0.929033637 | 5.04E-09 |
| LAMP3+ DC | EEF1A1 | 2.15E-11 | 0.829662204 | 1.099E-08 |
| LAMP3+ DC | MT-ND4L | 3E-11 | 0.79827863 | 1.375E-08 |
| LAMP3+ DC | RPS27 | 4.21E-11 | 0.725014806 | 1.85E-08 |
| LAMP3+ DC | RPS28 | 4.47E-11 | 0.550162613 | 1.93E-08 |
| LAMP3+ DC | PTMA | 9.07E-11 | 0.418745637 | 3.562E-08 |
| LAMP3+ DC | RPL36 | 9.21E-11 | 0.477515608 | 3.562E-08 |
| LAMP3+ DC | RPS8 | 1.26E-10 | 0.58488971 | 4.799E-08 |
| LAMP3+ DC | RPS15A | 5.61E-10 | 0.563026428 | 1.879E-07 |
| LAMP3+ DC | H3F3B | 7.81E-10 | 0.391081452 | 2.502E-07 |
| LAMP3+ DC | RPS3A | 1.37E-09 | 0.494040638 | 4.206E-07 |
| LAMP3+ DC | RPS29 | 5.33E-09 | 0.441179752 | 1.358E-06 |
| MTC CM | CD52 | 0 | 2.351050615 | 0 |
| MTC CM | IL32 | 0 | 2.892216921 | 0 |
| MTC CM | S100A10 | 0 | 1.966946721 | 0 |
| MTC CM | CRIP1 | 0 | 2.835954905 | 0 |
| MTC CM | TMSB10 | 0 | 1.520302773 | 0 |
| MTC CM | IFITM1 | 0 | 2.144979239 | 0 |
| MTC CM | B2M | 0 | 1.672727704 | 0 |
| MTC CM | TRBV7-2 | 0 | 4.897675991 | 0 |
| MTC CM | CD3D | 0 | 2.129636526 | 0 |
| MTC CM | LIMD2 | 0 | 1.467060328 | 0 |
| MTC CM | LTB | 0 | 2.167132616 | 0 |
| MTC CM | TMSB4X | 0 | 1.435465932 | 0 |
| MTC CM | RPS4X | 0 | 1.313147664 | 0 |
| MTC CM | HINT1 | 0 | 1.206465602 | 0 |
| MTC CM | PTPRCAP | 0 | 2.380532503 | 0 |
| MTC CM | MT-CO2 | 0 | 1.27576685 | 0 |
| MTC CM | LAT | 0 | 2.255853176 | 0 |
| MTC CM | NME2 | 0 | 1.949710846 | 0 |
| MTC CM | RPS16 | 0 | 0.920181572 | 0 |
| MTC CM | RPL17 | 0 | 1.478845596 | 0 |
| MTC CM | ACTG1 | 0 | 1.032556176 | 0 |
| MTC CM | RPS7 | 0 | 0.96927774 | 0 |
| MTC CM | CD99 | 0 | 1.658320785 | 0 |
| MTC CM | TAGLN2 | 0 | 1.190759182 | 0 |
| MTC CM | RPL18A | 0 | 1.127559543 | 0 |
| MTC E/EM | MTRNR2L12 | 0 | 3.797419786 | 0 |
| MTC E/EM | JUN | 0 | 3.159010172 | 0 |
| MTC E/EM | MALAT1 | 0 | 1.876743913 | 0 |
| MTC E/EM | BTG1 | 0 | 1.411910534 | 0 |
| MTC E/EM | JUNB | 0 | 1.717103004 | 0 |
| MTC E/EM | UBC | 0 | 1.393598676 | 0 |
| MTC E/EM | IER2 | 1.1E-281 | 1.934652567 | 2.75E-278 |
| MTC E/EM | PPP1R15A | 5.4E-274 | 2.62601757 | 1.2E-270 |
| MTC E/EM | H3F3B | 2.8E-269 | 1.076944113 | 5.69E-266 |
| MTC E/EM | MT-ATP6 | 4.2E-264 | 1.064448118 | 7.8E-261 |
| MTC E/EM | PTMA | 4.8E-249 | 0.833797872 | 7.17E-246 |
| MTC E/EM | HSP90AB1 | 8E-249 | 1.582884073 | 1.13E-245 |
| MTC E/EM | LMNA | 2.9E-247 | 3.13697052 | 3.88E-244 |
| MTC E/EM | NFKBIA | 1.4E-245 | 2.136007309 | 1.71E-242 |
| MTC E/EM | MT-CYB | 1.1E-239 | 0.937195718 | 1.3E-236 |
| MTC E/EM | HSP90AA1 | 4.3E-236 | 1.369690418 | 4.77E-233 |
| MTC E/EM | KLF6 | 1.3E-226 | 1.551624298 | 1.34E-223 |
| MTC E/EM | DDX5 | 5.4E-201 | 0.826834798 | 4.89E-198 |
| MTC E/EM | FOSB | 7.6E-198 | 3.108406305 | 6.59E-195 |
| MTC E/EM | EIF1 | 8.7E-190 | 0.683790624 | 7.27E-187 |
| MTC E/EM | GADD45B | 1.2E-180 | 2.795935869 | 8.7E-178 |
| MTC E/EM | BRD2 | 2.6E-177 | 2.16755271 | 1.84E-174 |
| MTC E/EM | MT-ND4L | 8.2E-177 | 1.052663088 | 5.57E-174 |
| MTC E/EM | NR4A2 | 1.2E-173 | 3.062298059 | 7.97E-171 |
| MTC E/EM | SRSF7 | 5.6E-170 | 1.784582257 | 3.42E-167 |
| MTC Reg | RPL23A | 3.6E-149 | 1.066788673 | 8.03E-145 |
| MTC Reg | MTRNR2L12 | 1.3E-142 | 2.830509901 | 1.5E-138 |
| MTC Reg | RPL41 | 9.8E-140 | 1.193085551 | 7.3E-136 |
| MTC Reg | LMNA | 4.4E-135 | 2.822323084 | 2.46E-131 |
| MTC Reg | VIM | 5.4E-116 | 1.058030844 | 2.44E-112 |
| MTC Reg | TRBC1 | 1.1E-112 | 1.977428436 | 4.3E-109 |
| MTC Reg | RGCC | 1.4E-112 | 2.283359051 | 4.5E-109 |
| MTC Reg | ITGB1 | 8.7E-107 | 1.341950178 | 2.43E-103 |
| MTC Reg | EIF1 | 7.8E-106 | 0.697488248 | 1.94E-102 |
| MTC Reg | H3F3B | 7.7E-104 | 0.776537478 | 1.62E-100 |
| MTC Reg | RPL19 | 1.6E-100 | 0.875975609 | 2.988E-97 |
| MTC Reg | PTMA | 2.7E-100 | 0.73281008 | 4.61E-97 |
| MTC Reg | MALAT1 | 6.3E-99 | 1.298456192 | 1.011E-95 |
| MTC Reg | BTG1 | 5.88E-94 | 0.948036194 | 8.79E-91 |
| MTC Reg | TSHZ2 | 1.42E-93 | 1.918074965 | 1.984E-90 |
| MTC Reg | HLA-A | 1.38E-91 | 0.77121067 | 1.826E-88 |
| MTC Reg | RPS21 | 4.82E-88 | 0.774923742 | 6.013E-85 |
| MTC Reg | RNF19A | 3.89E-87 | 1.995276809 | 4.591E-84 |
| MTC Reg | CCR7 | 4.74E-85 | 1.570336938 | 5.312E-82 |
| MTC Reg | BIRC3 | 1.24E-84 | 1.797584891 | 1.326E-81 |
| MTC Reg | UBC | 2.27E-80 | 1.08375001 | 2.126E-77 |
| MTC Reg | DUSP4 | 2.22E-74 | 3.270547628 | 1.917E-71 |
| MTC Reg | EEF1A1 | 2.24E-72 | 0.985729277 | 1.795E-69 |
| MTC Reg | PLS3 | 8.27E-72 | 2.930764437 | 6.395E-69 |
| MTC Reg | RPL38 | 9.46E-70 | 0.570886791 | 7.078E-67 |
| MP | GPX1 | 4.2E-237 | 3.447975397 | 9.44E-233 |
| MP | CTSB | 8.8E-219 | 2.760019541 | 9.83E-215 |
| MP | MS4A6A | 9.4E-203 | 3.228405476 | 7.05E-199 |
| MP | CD163 | 5.9E-201 | 3.605788946 | 3.32E-197 |
| MP | BLVRB | 1.8E-172 | 2.970825911 | 4.41E-169 |
| MP | ZYX | 1.7E-169 | 2.308404446 | 3.71E-166 |
| MP | GABARAP | 1.6E-166 | 2.213089228 | 2.7E-163 |
| MP | HMOX1 | 4E-164 | 3.77559495 | 6.4E-161 |
| MP | LST1 | 7.9E-161 | 2.646500349 | 1.11E-157 |
| MP | SPI1 | 3.8E-160 | 2.763626337 | 5.07E-157 |
| MP | STXBP2 | 1.7E-158 | 2.69820118 | 2.08E-155 |
| MP | AIF1 | 4.2E-157 | 2.640525818 | 4.91E-154 |
| MP | PGD | 1.1E-155 | 2.821619511 | 1.2E-152 |
| MP | TYMP | 1.1E-155 | 2.450615406 | 1.2E-152 |
| MP | FGR | 3.3E-155 | 2.715561867 | 3.32E-152 |
| MP | NPC2 | 6.6E-154 | 2.17496419 | 6.43E-151 |
| MP | IFI30 | 2.7E-152 | 4.126478672 | 2.51E-149 |
| MP | PSAP | 5E-152 | 1.884925008 | 4.53E-149 |
| MP | RNF130 | 5.1E-151 | 2.830433607 | 4.4E-148 |
| MP | PYCARD | 4.7E-150 | 2.258198023 | 3.92E-147 |
| MP | VCAN | 4E-147 | 2.791413069 | 3.09E-144 |
| MP | SULT1A1 | 1.3E-146 | 3.596722603 | 9.48E-144 |
| MP | C1orf162 | 5.5E-146 | 2.302686214 | 3.97E-143 |
| MP | CST3 | 1.4E-144 | 2.043902636 | 9.61E-142 |
| MP | LILRB2 | 1.4E-144 | 3.395792961 | 9.69E-142 |
| ML Mono | LYZ | 1.6E-165 | 4.437789917 | 3.5E-161 |
| ML Mono | S100A9 | 1.3E-161 | 5.042582035 | 1.4E-157 |
| ML Mono | S100A8 | 4.3E-159 | 5.525038719 | 3.19E-155 |
| ML Mono | FTL | 2.5E-157 | 3.278684378 | 1.38E-153 |
| ML Mono | TYROBP | 4.9E-156 | 3.785999298 | 2.21E-152 |
| ML Mono | CST3 | 2.2E-155 | 4.109960079 | 8.25E-152 |
| ML Mono | FTH1 | 2.2E-143 | 2.407633066 | 7.14E-140 |
| ML Mono | FCN1 | 2.8E-138 | 3.503867388 | 7.83E-135 |
| ML Mono | S100A6 | 6.9E-134 | 2.193773031 | 1.72E-130 |
| ML Mono | PSAP | 3.2E-133 | 2.696703911 | 7.09E-130 |
| ML Mono | CTSS | 1.2E-131 | 2.842621565 | 2.41E-128 |
| ML Mono | AIF1 | 2.3E-126 | 3.121240854 | 4.22E-123 |
| ML Mono | LST1 | 9.3E-122 | 2.885006428 | 1.6E-118 |
| ML Mono | TSPO | 2E-121 | 2.106232405 | 3.13E-118 |
| ML Mono | GABARAP | 2.8E-120 | 2.308771133 | 4.13E-117 |
| ML Mono | OAZ1 | 8.2E-117 | 1.504693747 | 1.14E-113 |
| ML Mono | VCAN | 8.3E-109 | 3.049759865 | 1.1E-105 |
| ML Mono | CD74 | 5.4E-108 | 2.689528704 | 6.69E-105 |
| ML Mono | SAT1 | 3.3E-107 | 2.356158733 | 3.86E-104 |
| ML Mono | GSTP1 | 4.1E-106 | 2.298722982 | 4.58E-103 |
| ML Mono | NCF1 | 2.2E-104 | 3.123024702 | 2.3E-101 |
| ML Mono | S100A4 | 6.8E-104 | 1.915719271 | 6.93E-101 |
| ML Mono | S100A12 | 7.7E-104 | 3.082556963 | 7.51E-101 |
| ML Mono | NEAT1 | 6E-103 | 2.091389894 | 5.64E-100 |
| ML Mono | SPI1 | 1.89E-96 | 2.582244396 | 1.692E-93 |
| Memory B | CD79A | 0 | 5.871738911 | 0 |
| Memory B | MS4A1 | 0 | 6.023410797 | 0 |
| Memory B | BANK1 | 0 | 6.018186569 | 0 |
| Memory B | CD79B | 0 | 4.773616314 | 0 |
| Memory B | RALGPS2 | 0 | 5.342929363 | 0 |
| Memory B | HLA-DPB1 | 0 | 2.990559101 | 0 |
| Memory B | TNFRSF13C | 2.4E-302 | 6.182079315 | 7.78E-299 |
| Memory B | HLA-DPA1 | 7.4E-283 | 2.702003956 | 1.67E-279 |
| Memory B | HLA-DRA | 3.1E-277 | 2.852632284 | 6.42E-274 |
| Memory B | LINC00926 | 5.5E-269 | 5.868813038 | 9.41E-266 |
| Memory B | IGHM | 1.4E-267 | 5.162686825 | 2.2E-264 |
| Memory B | FCMR | 1.5E-267 | 3.009572268 | 2.22E-264 |
| Memory B | IGKC | 2.9E-262 | 4.158593178 | 4.06E-259 |
| Memory B | HLA-DQB1 | 6.7E-255 | 3.774262905 | 8.78E-252 |
| Memory B | BLK | 1.4E-243 | 6.172082901 | 1.65E-240 |
| Memory B | IRF8 | 1.8E-237 | 3.750537872 | 2.04E-234 |
| Memory B | HLA-DMA | 2.5E-236 | 3.137788773 | 2.67E-233 |
| Memory B | POU2F2 | 1.3E-226 | 2.672762632 | 1.29E-223 |
| Memory B | CD22 | 6.1E-221 | 6.092372894 | 5.94E-218 |
| Memory B | HLA-DRB1 | 3E-218 | 2.623134851 | 2.79E-215 |
| Memory B | HLA-DMB | 2.5E-216 | 3.579456329 | 2.25E-213 |
| Memory B | SWAP70 | 6.5E-206 | 4.034497261 | 5.02E-203 |
| Memory B | BCL11A | 2.9E-202 | 5.168663025 | 2.13E-199 |
| Memory B | HLA-DQA1 | 1E-201 | 3.910999537 | 7.19E-199 |
| Memory B | MEF2C | 3.4E-196 | 3.179704666 | 2.21E-193 |
| NK | NKG7 | 0 | 5.04956913 | 0 |
| NK | PRF1 | 0 | 4.82682991 | 0 |
| NK | GZMB | 0 | 5.337763309 | 0 |
| NK | CTSW | 0 | 4.419467449 | 0 |
| NK | GZMA | 0 | 4.496548176 | 0 |
| NK | KLRD1 | 0 | 4.898091793 | 0 |
| NK | GNLY | 0 | 5.039386749 | 0 |
| NK | CST7 | 0 | 3.344718695 | 0 |
| NK | HOPX | 0 | 4.2488451 | 0 |
| NK | FGFBP2 | 0 | 4.879288673 | 0 |
| NK | CD7 | 0 | 3.274960041 | 0 |
| NK | CCL5 | 0 | 3.960488558 | 0 |
| NK | KLRF1 | 0 | 5.63166666 | 0 |
| NK | CD247 | 0 | 2.65647459 | 0 |
| NK | FCGR3A | 0 | 3.573628187 | 0 |
| NK | GZMM | 0 | 3.331202745 | 0 |
| NK | CLIC3 | 0 | 5.074412346 | 0 |
| NK | SPON2 | 0 | 4.693784237 | 0 |
| NK | KLRB1 | 0 | 3.660100222 | 0 |
| NK | HCST | 0 | 1.836464763 | 0 |
| NK | CCL4 | 0 | 3.719687939 | 0 |
| NK | IL2RB | 0 | 3.737388134 | 0 |
| NK | PLAC8 | 0 | 2.48375988 | 0 |
| NK | EFHD2 | 0 | 2.105691671 | 0 |
| NK | MATK | 0 | 4.152040005 | 0 |
| NK-Like Mono | S100A8 | 3.62E-57 | 5.276485443 | 8.121E-53 |
| NK-Like Mono | S100A9 | 6.07E-51 | 3.991076231 | 6.81E-47 |
| NK-Like Mono | FTL | 2.26E-48 | 2.70349431 | 1.688E-44 |
| NK-Like Mono | TYROBP | 3.31E-46 | 3.204213381 | 1.856E-42 |
| NK-Like Mono | CST3 | 8.59E-46 | 3.368080616 | 3.855E-42 |
| NK-Like Mono | LYZ | 1.12E-45 | 3.335815191 | 4.206E-42 |
| NK-Like Mono | FTH1 | 1.99E-45 | 2.116714239 | 6.393E-42 |
| NK-Like Mono | NEAT1 | 3.31E-41 | 2.069669008 | 9.293E-38 |
| NK-Like Mono | S100A6 | 5.23E-41 | 1.988019228 | 1.303E-37 |
| NK-Like Mono | FOS | 1.05E-35 | 2.520178795 | 2.366E-32 |
| NK-Like Mono | S100A4 | 2.47E-35 | 1.844208121 | 5.039E-32 |
| NK-Like Mono | GABARAP | 2.84E-35 | 2.025396585 | 5.311E-32 |
| NK-Like Mono | NKG7 | 3.4E-34 | 3.237766266 | 5.862E-31 |
| NK-Like Mono | S100A12 | 1.28E-33 | 2.78222394 | 2.051E-30 |
| NK-Like Mono | GSTP1 | 1.09E-32 | 2.125247717 | 1.634E-29 |
| NK-Like Mono | CCL5 | 4.08E-32 | 3.155453682 | 5.716E-29 |
| NK-Like Mono | FCN1 | 3.17E-31 | 2.59732008 | 4.178E-28 |
| NK-Like Mono | CD74 | 3.41E-31 | 1.945868254 | 4.248E-28 |
| NK-Like Mono | VCAN | 7.81E-30 | 2.521843195 | 9.225E-27 |
| NK-Like Mono | AIF1 | 4.82E-29 | 2.328007698 | 5.407E-26 |
| NK-Like Mono | GNLY | 9.42E-29 | 3.510245085 | 1.007E-25 |
| NK-Like Mono | MT-ND4 | 1.09E-27 | 1.176753879 | 1.112E-24 |
| NK-Like Mono | NCF1 | 2.26E-27 | 2.342805624 | 2.201E-24 |
| NK-Like Mono | TSPO | 8.63E-26 | 1.510815024 | 8.069E-23 |
| NK-Like Mono | MTRNR2L8 | 1.28E-25 | 3.490778923 | 1.147E-22 |
| Naive B | CD79A | 0 | 7.416737556 | 0 |
| Naive B | MS4A1 | 0 | 7.727255821 | 0 |
| Naive B | HLA-DPB1 | 0 | 3.800414562 | 0 |
| Naive B | HLA-DRA | 0 | 4.030269146 | 0 |
| Naive B | HLA-DPA1 | 0 | 3.649439812 | 0 |
| Naive B | CD79B | 0 | 4.754781723 | 0 |
| Naive B | CD37 | 0 | 2.156935692 | 0 |
| Naive B | BANK1 | 0 | 6.415452957 | 0 |
| Naive B | IGHM | 0 | 5.95330286 | 0 |
| Naive B | HLA-DQB1 | 0 | 4.348500729 | 0 |
| Naive B | LINC00926 | 0 | 7.004324913 | 0 |
| Naive B | HLA-DQA1 | 0 | 4.978325844 | 0 |
| Naive B | HLA-DRB1 | 0 | 3.46587348 | 0 |
| Naive B | CD74 | 0 | 3.659224033 | 0 |
| Naive B | RALGPS2 | 0 | 5.222727776 | 0 |
| Naive B | IGHD | 0 | 7.307136536 | 0 |
| Naive B | MEF2C | 0 | 3.433150768 | 0 |
| Naive B | HLA-DMA | 0 | 3.02235961 | 0 |
| Naive B | FCMR | 0 | 2.67693615 | 0 |
| Naive B | CXCR4 | 0 | 2.157906771 | 0 |
| Naive B | FAM129C | 0 | 6.644666195 | 0 |
| Naive B | FCER2 | 0 | 6.956933022 | 0 |
| Naive B | CD22 | 0 | 6.540520191 | 0 |
| Naive B | IRF8 | 0 | 3.325276136 | 0 |
| Naive B | BLK | 0 | 6.129780293 | 0 |
| NC Mono | LST1 | 0 | 4.560498238 | 0 |
| NC Mono | PSAP | 0 | 3.772663116 | 0 |
| NC Mono | AIF1 | 0 | 4.217755318 | 0 |
| NC Mono | IFITM3 | 0 | 4.602684975 | 0 |
| NC Mono | FCER1G | 0 | 4.019050598 | 0 |
| NC Mono | FTH1 | 0 | 3.269052982 | 0 |
| NC Mono | SERPINA1 | 0 | 4.255123615 | 0 |
| NC Mono | SAT1 | 0 | 3.396457195 | 0 |
| NC Mono | FCGR3A | 0 | 4.565264225 | 0 |
| NC Mono | FTL | 0 | 3.651969433 | 0 |
| NC Mono | CST3 | 0 | 4.525194168 | 0 |
| NC Mono | HLA-DPA1 | 0 | 3.925172091 | 0 |
| NC Mono | TYROBP | 0 | 3.863112211 | 0 |
| NC Mono | MS4A7 | 0 | 4.153521061 | 0 |
| NC Mono | CD68 | 0 | 4.051233292 | 0 |
| NC Mono | CTSS | 0 | 3.138445616 | 0 |
| NC Mono | CD74 | 0 | 3.626972198 | 0 |
| NC Mono | S100A11 | 0 | 2.319823265 | 0 |
| NC Mono | SPI1 | 0 | 3.31670022 | 0 |
| NC Mono | HLA-DRB1 | 0 | 3.695339441 | 0 |
| NC Mono | CFD | 0 | 3.742064476 | 0 |
| NC Mono | COTL1 | 0 | 2.517810583 | 0 |
| NC Mono | HLA-DRA | 0 | 3.67037034 | 0 |
| NC Mono | OAZ1 | 0 | 1.706267595 | 0 |
| NC Mono | IFITM2 | 0 | 2.33959341 | 0 |
| T CM | RPS12 | 0 | 1.523334026 | 0 |
| T CM | RPL13 | 0 | 1.322485685 | 0 |
| T CM | RPL34 | 0 | 1.242499948 | 0 |
| T CM | EEF1A1 | 0 | 1.362612009 | 0 |
| T CM | RPL32 | 0 | 1.262604237 | 0 |
| T CM | RPS6 | 0 | 1.282004833 | 0 |
| T CM | RPS27 | 0 | 1.43744266 | 0 |
| T CM | RPS27A | 0 | 1.205642939 | 0 |
| T CM | RPS3A | 0 | 1.23378861 | 0 |
| T CM | RPS25 | 0 | 1.222782731 | 0 |
| T CM | RPS29 | 0 | 1.435167789 | 0 |
| T CM | RPL3 | 0 | 1.277033925 | 0 |
| T CM | RPS8 | 0 | 1.227128506 | 0 |
| T CM | RPL35A | 0 | 1.103004336 | 0 |
| T CM | RPS3 | 0 | 1.193499327 | 0 |
| T CM | RPL37 | 0 | 1.113551021 | 0 |
| T CM | RPL39 | 0 | 1.183073163 | 0 |
| T CM | RPL10A | 0 | 1.16237855 | 0 |
| T CM | RPS18 | 0 | 1.292509198 | 0 |
| T CM | RPL9 | 0 | 1.131461859 | 0 |
| T CM | RPL30 | 0 | 1.176509142 | 0 |
| T CM | RPL36 | 0 | 1.123114586 | 0 |
| T CM | RPS23 | 0 | 1.158531547 | 0 |
| T CM | RPL5 | 0 | 1.127849936 | 0 |
| T CM | RPL11 | 0 | 1.099228859 | 0 |
| T EM | PFN1 | 0 | 0.986687362 | 0 |
| T EM | IL32 | 0 | 1.610342145 | 0 |
| T EM | CD3E | 1.3E-304 | 1.308663845 | 9.47E-301 |
| T EM | CD2 | 1.1E-287 | 1.467312932 | 6.15E-284 |
| T EM | LCK | 2.6E-272 | 1.262953758 | 1.17E-268 |
| T EM | RPSA | 4.4E-253 | 0.814624369 | 1.66E-249 |
| T EM | CALM1 | 7.9E-253 | 0.715068698 | 2.52E-249 |
| T EM | ARHGDIB | 4.6E-251 | 0.718464315 | 1.29E-247 |
| T EM | CNN2 | 1.9E-232 | 1.090877652 | 4.77E-229 |
| T EM | PPDPF | 1.9E-221 | 0.780041814 | 4.36E-218 |
| T EM | HMGB1 | 2.1E-220 | 0.805478156 | 4.24E-217 |
| T EM | C12orf75 | 9.5E-216 | 1.496819973 | 1.78E-212 |
| T EM | GZMA | 1.5E-208 | 1.859913707 | 2.62E-205 |
| T EM | CFL1 | 2.8E-208 | 0.597434163 | 4.46E-205 |
| T EM | GAPDH | 8.5E-204 | 0.773029089 | 1.28E-200 |
| T EM | HNRNPA1 | 2E-199 | 0.635649562 | 2.79E-196 |
| T EM | CD3D | 1.6E-196 | 1.050539613 | 2.15E-193 |
| T EM | HSPA8 | 8.1E-196 | 0.768334746 | 1.01E-192 |
| T EM | PPIA | 1.4E-193 | 0.677782595 | 1.6E-190 |
| T EM | CORO1A | 3.3E-193 | 0.678872168 | 3.75E-190 |
| T EM | TMSB4X | 3E-192 | 0.701423883 | 3.18E-189 |
| T EM | RPLP0 | 5.7E-191 | 0.735651016 | 5.82E-188 |
| T EM | NPM1 | 4.2E-189 | 0.708239377 | 4.14E-186 |
| T EM | CD3G | 2.1E-188 | 1.052509427 | 1.94E-185 |
| T EM | RPL35 | 3.8E-183 | 0.589234889 | 3.32E-180 |
| T Reg | TRAC | 2.1E-186 | 2.504023075 | 4.74E-182 |
| T Reg | CD2 | 9.6E-168 | 2.014061689 | 1.08E-163 |
| T Reg | LCK | 9.8E-150 | 1.721353292 | 7.33E-146 |
| T Reg | BCL11B | 1.9E-147 | 2.586610556 | 1.05E-143 |
| T Reg | SPOCK2 | 1.4E-142 | 1.985653996 | 6.27E-139 |
| T Reg | PRDX2 | 1.2E-141 | 1.983315706 | 4.59E-138 |
| T Reg | ACAP1 | 2.4E-138 | 1.722847581 | 7.82E-135 |
| T Reg | CD3G | 5.5E-135 | 1.680864692 | 1.54E-131 |
| T Reg | TRAF3IP3 | 2.2E-134 | 1.42104578 | 5.38E-131 |
| T Reg | RCAN3 | 1.1E-121 | 2.344667435 | 2.41E-118 |
| T Reg | NOSIP | 1.2E-116 | 1.687200785 | 2.38E-113 |
| T Reg | LINC00861 | 1.6E-114 | 2.534571886 | 2.99E-111 |
| T Reg | ETS1 | 8.6E-107 | 1.445593357 | 1.48E-103 |
| T Reg | LINC-PINT | 2.1E-106 | 2.737786531 | 3.31E-103 |
| T Reg | CD48 | 2.7E-106 | 1.103573203 | 4.1E-103 |
| T Reg | AAK1 | 7.8E-106 | 1.541362524 | 1.09E-102 |
| T Reg | LDHB | 4.4E-103 | 1.221625805 | 5.75E-100 |
| T Reg | SEPT1 | 7.4E-103 | 1.73498702 | 9.17E-100 |
| T Reg | RPS20 | 3.5E-102 | 0.99298352 | 4.13E-99 |
| T Reg | BIN1 | 8E-102 | 1.613268495 | 8.99E-99 |
| T Reg | MZT2B | 2.9E-101 | 1.265387058 | 3.115E-98 |
| T Reg | LEPROTL1 | 1.15E-97 | 1.378185511 | 1.178E-94 |
| T Reg | SEPT6 | 7.29E-96 | 1.290680408 | 7.112E-93 |
| T Reg | PIK3IP1 | 1.29E-95 | 1.614526391 | 1.203E-92 |
| T Reg | EVL | 3.22E-95 | 1.193714261 | 2.886E-92 |
| Tn | CD7 | 2.4E-145 | 1.697336078 | 8.8E-142 |
| Tn | ETS1 | 6.76E-92 | 1.095367551 | 1.011E-88 |
| Tn | PIK3IP1 | 1.82E-81 | 1.30270195 | 1.948E-78 |
| Tn | LINC00861 | 6.47E-80 | 1.70900321 | 6.601E-77 |
| Tn | TXK | 1.56E-77 | 2.393122911 | 1.524E-74 |
| Tn | OXNAD1 | 9.05E-76 | 2.057674646 | 8.458E-73 |
| Tn | TXNIP | 2.58E-75 | 0.686633885 | 2.319E-72 |
| Tn | CXCR4 | 2.02E-70 | 0.973678648 | 1.679E-67 |
| Tn | CD69 | 8.34E-68 | 0.95116365 | 6.451E-65 |
| Tn | RPS20 | 1.69E-66 | 0.62431258 | 1.266E-63 |
| Tn | SLC38A1 | 2.29E-66 | 1.239191532 | 1.659E-63 |
| Tn | STK17A | 1.48E-63 | 1.099060893 | 9.793E-61 |
| Tn | PDCD4 | 7.3E-62 | 1.224786997 | 4.552E-59 |
| Tn | ACAP1 | 2.05E-60 | 0.978610933 | 1.244E-57 |
| Tn | CD2 | 8.5E-58 | 1.040145159 | 4.653E-55 |
| Tn | FCMR | 3.39E-56 | 1.117510676 | 1.812E-53 |
| Tn | GCC2 | 3.5E-53 | 1.097834826 | 1.634E-50 |
| Tn | ATM | 2.73E-52 | 1.01020813 | 1.201E-49 |
| Tn | NOSIP | 7.53E-52 | 0.947219908 | 3.189E-49 |
| Tn | RPL27A | 1.15E-51 | 0.482233763 | 4.596E-49 |
| Tn | SNHG8 | 2.9E-51 | 0.827898383 | 1.14E-48 |
| Tn | PSIP1 | 6.3E-50 | 1.007369518 | 2.318E-47 |
| Tn | PNISR | 6.88E-50 | 0.649157584 | 2.488E-47 |
| Tn | NPM1 | 2.42E-49 | 0.534767032 | 8.495E-47 |
| Tn | TOMM7 | 2.7E-49 | 0.523479581 | 9.325E-47 |
| cDC2 | CD74 | 2.7E-207 | 5.057557583 | 6.09E-203 |
| cDC2 | HLA-DRA | 2.5E-193 | 4.824946404 | 2.79E-189 |
| cDC2 | HLA-DRB1 | 2.6E-189 | 4.507218361 | 1.96E-185 |
| cDC2 | HLA-DPA1 | 9.1E-185 | 4.348133564 | 5.09E-181 |
| cDC2 | HLA-DPB1 | 5.8E-183 | 4.203994751 | 2.6E-179 |
| cDC2 | CST3 | 1.3E-174 | 4.506518364 | 4.92E-171 |
| cDC2 | HLA-DRB5 | 2.5E-161 | 4.534143925 | 7.95E-158 |
| cDC2 | HLA-DMA | 3.5E-156 | 3.360064745 | 9.95E-153 |
| cDC2 | HLA-DQA1 | 4.9E-154 | 4.728191853 | 1.21E-150 |
| cDC2 | HLA-DQB1 | 1.9E-140 | 4.238793373 | 4.31E-137 |
| cDC2 | FTH1 | 1E-131 | 1.777403235 | 2.03E-128 |
| cDC2 | PLD4 | 9E-124 | 6.3570261 | 1.68E-120 |
| cDC2 | GSTP1 | 1.4E-121 | 2.289432287 | 2.39E-118 |
| cDC2 | FCER1G | 3.3E-109 | 2.372119427 | 5.26E-106 |
| cDC2 | GABARAP | 4.6E-105 | 1.864873886 | 6.89E-102 |
| cDC2 | TYROBP | 5.9E-105 | 2.443982363 | 8.23E-102 |
| cDC2 | CYBA | 1.3E-100 | 1.42224133 | 1.757E-97 |
| cDC2 | GRN | 2.63E-94 | 2.188431263 | 3.282E-91 |
| cDC2 | GSN | 1.12E-89 | 2.682803154 | 1.317E-86 |
| cDC2 | FTL | 2.74E-85 | 1.364216805 | 2.954E-82 |
| cDC2 | RPLP0 | 2.77E-85 | 1.221267819 | 2.954E-82 |
| cDC2 | HLA-DMB | 3.07E-84 | 2.364899874 | 3.127E-81 |
| cDC2 | FCER1A | 2.49E-82 | 8.013721466 | 2.424E-79 |
| cDC2 | LSP1 | 7.61E-82 | 1.397130966 | 7.112E-79 |
| cDC2 | CAPG | 4.76E-81 | 1.980050206 | 4.268E-78 |
| pDC | PRSS57 | 6.59E-57 | 8.385089874 | 3.694E-53 |
| pDC | SOX4 | 1.53E-55 | 4.120506287 | 4.3E-52 |
| pDC | CDK6 | 3.4E-55 | 4.977421761 | 8.472E-52 |
| pDC | LAPTM4B | 4.31E-50 | 7.235749722 | 8.8E-47 |
| pDC | EBPL | 4.74E-50 | 3.683744431 | 8.866E-47 |
| pDC | SMIM24 | 1.04E-48 | 10.21585178 | 1.789E-45 |
| pDC | SNHG7 | 1.55E-48 | 2.896008968 | 2.479E-45 |
| pDC | STMN1 | 6.19E-48 | 3.380157232 | 8.244E-45 |
| pDC | TSC22D1 | 6.25E-48 | 4.35357666 | 8.244E-45 |
| pDC | ANKRD28 | 4.03E-47 | 3.901785135 | 4.764E-44 |
| pDC | AMD1 | 3.37E-46 | 2.746589661 | 3.775E-43 |
| pDC | NUCB2 | 4.16E-46 | 3.128644466 | 4.449E-43 |
| pDC | IMPDH2 | 5.53E-46 | 3.348272324 | 5.636E-43 |
| pDC | ZNRF1 | 1.15E-44 | 4.666395187 | 1.075E-41 |
| pDC | HMGA1 | 3.85E-44 | 2.729388952 | 3.318E-41 |
| pDC | DDAH2 | 6.83E-44 | 3.38551259 | 5.675E-41 |
| pDC | SSBP2 | 7.58E-44 | 3.770020962 | 6E-41 |
| pDC | SPINT2 | 2.12E-42 | 2.846805096 | 1.535E-39 |
| pDC | BEX4 | 8.66E-42 | 3.606184006 | 6.071E-39 |
| pDC | EGFL7 | 3.82E-41 | 7.544454575 | 2.523E-38 |
| pDC | BEX2 | 1.28E-40 | 4.042181492 | 7.962E-38 |
| pDC | APEX1 | 3.83E-40 | 2.435176611 | 2.322E-37 |
| pDC | BZW2 | 7.5E-40 | 3.644649267 | 4.426E-37 |
| pDC | KDM5B | 8.14E-40 | 3.443030596 | 4.68E-37 |
| pDC | MYB | 1.57E-39 | 6.873373032 | 8.539E-37 |

**Table S4. Differentially expressed genes in each cell cluster**

The top 25 differentially expressed genes (DEGs) per cluster were ranked by log_2_ fold change. DEGs with an adjusted p-value<0.05 using the Wilcoxon Rank Sum Test with Bonferroni correction were selected. *Abbreviations: CD8+ T, cytotoxic CD8+ T cells; Class Mono, classical monocytes; Int Mono, intermediate monocytes; DC, dendritic cells; MTC, malignant T cells; CM, central memory; E/EM, effector/memory; Reg, regulatory; MP, macrophage; ML Mono, megakaryocyte-like monocytes; B, B cells; NK, natural killer cells; NK-Like Mono, natura killer cell-like monocytes; NC Mono, nonclassical monocytes; Tn, T-naïve cells; cDC2, type 2 conventional dendritic cells; pDC, plasmacytoid dendritic cells.*

| **gene** | **qval** | **log2fc** | **mean** |
| --- | --- | --- | --- |
| HACD1 | 0 | 2.509863 | 0.113629 |
| PLS3 | 0 | 2.44166 | 0.095377 |
| CD70 | 0 | 2.177189 | 0.122666 |
| NEDD4L | 0 | 1.94043 | 0.233707 |
| KIR3DL2 | 0 | 1.886088 | 0.103778 |
| TPO | 0 | 1.767456 | 0.096776 |
| IGFBP4 | 0 | 1.750654 | 0.202557 |
| PTHLH | 0 | 1.739237 | 0.071008 |
| LAIR2 | 0 | 1.727247 | 0.073169 |
| COL6A2 | 0 | 1.640846 | 0.107552 |
| CXCL13 | 0 | 1.609768 | 0.098866 |
| DUSP4 | 0 | 1.56176 | 0.108303 |
| AIRE | 0 | 1.49677 | 0.520241 |
| KLHL42 | 0 | 1.486158 | 0.137819 |
| CHN1 | 0 | 1.457556 | 0.059486 |
| LMNA | 0 | 1.456174 | 0.296649 |
| GNG4 | 0 | 1.448043 | 0.073369 |
| PDLIM1 | 0 | 1.425236 | 0.233056 |
| PTMS | 0 | 1.412676 | 0.179403 |
| TUSC3 | 0 | 1.40849 | 0.110564 |
| TSPAN2 | 0 | 1.402891 | 0.160644 |
| DNM3OS | 0 | 1.389316 | 0.081069 |
| CCR4 | 0 | 1.3837 | 0.166911 |
| CCR10 | 0 | 1.380784 | 0.07106 |
| CX3CR1 | 0 | 1.327656 | 0.081138 |
| PCED1B | 0 | -2.2466 | 0.051238 |
| ITGA4 | 0 | -2.01783 | 0.066787 |
| PLAC8 | 0 | -1.86594 | 0.061499 |
| SATB1 | 0 | -1.86033 | 0.068197 |
| CD7 | 0 | -1.55611 | 0.243773 |
| LINC00861 | 0 | -1.5198 | 0.142767 |
| OXNAD1 | 0 | -1.49084 | 0.105907 |
| C1orf162 | 0 | -1.4568 | 0.089908 |
| TRABD2A | 0 | -1.44754 | 0.054948 |
| NUCB2 | 0 | -1.33776 | 0.072825 |
| GSTP1 | 0 | -1.28612 | 0.078229 |
| UPP1 | 0 | -1.21085 | 0.054063 |
| ABLIM1 | 0 | -1.15278 | 0.114434 |
| PRDX2 | 0 | -1.14054 | 0.140578 |
| CHCHD10 | 0 | -1.12019 | 0.112606 |
| CISH | 0 | -1.09292 | 0.104363 |
| LINC01550 | 0 | -1.07082 | 0.055127 |
| IFI44L | 0 | -1.06992 | 0.051247 |
| PDCD4 | 0 | -0.99829 | 0.119053 |
| CD96 | 0 | -0.98633 | 0.091229 |
| APBB1IP | 0 | -0.9563 | 0.119896 |
| FOXP1 | 0 | -0.93934 | 0.173263 |
| KLRB1 | 0 | -0.88041 | 0.13266 |
| FHIT | 0 | -0.87461 | 0.059252 |
| XBP1 | 0 | -0.87376 | 0.118017 |

**Table S5. Differentially expressed genes in MTC CM vs. T CM**

The top 25 differentially upregulated and downregulated differentially expressed genes (DEGs) in malignant central memory T cells (MTC CM) compared to benign central memory T cells (T CM) in cutaneous T cell lymphoma (CTCL) patients were ranked by log2 fold change. DEGs with an adjusted p-value (q-value) <0.05, corrected for multiple testing using the Benjamini-Hochberg (FDR) procedure, were selected. The table includes the following columns: q-value (FDR-adjusted p-value), log2fc (log2 fold change), and mean (average expression).

| **gene** | **qval** | **log2fc** | **mean** |
| --- | --- | --- | --- |
| HSPA6 | 0 | 4.114767 | 0.14161 |
| RASD1 | 0 | 3.239503 | 0.059424 |
| NDUFA4L2 | 0 | 3.076283 | 0.062238 |
| NR4A1 | 0 | 2.945409 | 0.234997 |
| EGR1 | 0 | 2.944622 | 0.264574 |
| PLK2 | 0 | 2.845227 | 0.121721 |
| HSPA1B | 0 | 2.734522 | 0.305756 |
| HSPA2 | 0 | 2.727889 | 0.101437 |
| IER5L | 0 | 2.6568 | 0.051105 |
| PLCG2 | 0 | 2.648124 | 0.11248 |
| ATF3 | 0 | 2.518105 | 0.17083 |
| RASGEF1B | 0 | 2.409381 | 0.055284 |
| IER3 | 0 | 2.398943 | 0.16462 |
| FOSB | 0 | 2.391358 | 0.479218 |
| DNAJA4 | 0 | 2.381732 | 0.071297 |
| KCNQ1OT1 | 0 | 2.346339 | 0.137821 |
| HEXIM1 | 0 | 2.290243 | 0.336011 |
| TNFSF9 | 0 | 2.278614 | 0.080464 |
| GEM | 0 | 2.273945 | 0.079405 |
| AC007952.4 | 0 | 2.263094 | 0.1078 |
| HMOX1 | 0 | 2.226942 | 0.198073 |
| MAFF | 0 | 2.220741 | 0.075213 |
| CD83 | 0 | 2.126164 | 0.214394 |
| NR4A3 | 0 | 2.123712 | 0.141404 |
| ZNF331 | 0 | 2.106729 | 0.281252 |
| TYMS | 0 | -4.71252 | 0.125722 |
| RRM2 | 0 | -4.66424 | 0.114113 |
| UBE2C | 0 | -4.60012 | 0.079727 |
| BIRC5 | 0 | -3.71666 | 0.077269 |
| MKI67 | 0 | -3.51605 | 0.093071 |
| HIST1H1B | 0 | -3.46462 | 0.051442 |
| TK1 | 0 | -3.2547 | 0.06892 |
| TOP2A | 0 | -3.11743 | 0.115633 |
| CDT1 | 0 | -2.97545 | 0.053438 |
| TPX2 | 0 | -2.48347 | 0.056542 |
| MAD2L1 | 0 | -2.37071 | 0.060912 |
| TMEM256-PLSCR3 | 0 | -2.36854 | 0.075202 |
| CCR10 | 0 | -2.26829 | 0.115026 |
| DHFR | 0 | -2.22662 | 0.061298 |
| ASF1B | 0 | -2.13534 | 0.052278 |
| FUT7 | 0 | -2.07425 | 0.063562 |
| JAKMIP1 | 0 | -2.05731 | 0.061764 |
| MYCBP | 0 | -2.02889 | 0.066788 |
| CDCA7 | 0 | -1.97567 | 0.115203 |
| EEF1G | 0 | -1.91838 | 0.414177 |
| C11orf98 | 0 | -1.90265 | 0.093952 |
| SMCO4 | 0 | -1.83225 | 0.052236 |
| DTYMK | 0 | -1.73772 | 0.06692 |
| RFC4 | 0 | -1.70636 | 0.051465 |
| C12orf75 | 0 | -1.70115 | 0.290426 |

**Table S6. Differentially expressed genes in MTC E/EM vs. T EM**
The top 25 differentially upregulated and downregulated differentially expressed genes (DEGs) in malignant effector and effector memory T cells (MTC E/EM) compared to benign effector memory T cells (T EM) in cutaneous T cell lymphoma (CTCL) patients were ranked by log2 fold change. DEGs with an adjusted p-value (q-value) <0.05, corrected for multiple testing using the Benjamini-Hochberg (FDR) procedure, were selected. The table includes the following columns: q-value (FDR-adjusted p-value), log2fc (log2 fold change), and mean (average expression).

| **gene** | **qval** | **log2fc** | **mean** |
| --- | --- | --- | --- |
| CX3CR1 | 0 | 297.776 | 0.082452 |
| MLF1 | 0 | 297.776 | 0.130574 |
| TNFSF11 | 0 | 297.776 | 0.067457 |
| EGLN3 | 0 | 297.776 | 0.095328 |
| GEM | 0 | 297.776 | 0.135996 |
| KIR3DL2 | 0 | 297.776 | 0.111761 |
| DNM3OS | 0 | 297.776 | 0.08504 |
| EPCAM | 0 | 297.776 | 0.118209 |
| PLS3 | 0 | 297.776 | 0.284141 |
| HDAC9 | 0 | 297.776 | 0.064538 |
| TGFBR3 | 0.024745 | 3.309968 | 0.205909 |
| AC016831.7 | 0.017416 | 3.277908 | 0.221129 |
| CYSTM1 | 0.019446 | 2.924482 | 0.190796 |
| DUSP4 | 1.2E-05 | 2.81002 | 0.468909 |
| NR4A3 | 0.018509 | 2.754243 | 0.183999 |
| NEDD4L | 0.000919 | 2.745122 | 0.297949 |
| PTMS | 0.024745 | 2.466492 | 0.161381 |
| SLC5A3 | 0.012336 | 2.44824 | 0.188599 |
| CCR4 | 0.001048 | 2.370485 | 0.272235 |
| LMNA | 2.14E-12 | 2.364509 | 0.948709 |
| TSPAN2 | 0.01684 | 2.318517 | 0.173443 |
| IKZF2 | 0.007507 | 2.299237 | 0.203051 |
| CD70 | 0.018509 | 2.292651 | 0.168745 |
| ANKRD28 | 0.021699 | 2.255811 | 0.16163 |
| CDKN1A | 0.000973 | 2.214324 | 0.272257 |
| XAF1 | 0 | -1.80655 | 0.11718 |
| IFITM3 | 3.2E-12 | -1.66561 | 0.085332 |
| PLAC8 | 3.73E-08 | -1.59642 | 0.063804 |
| SATB1 | 4.3E-08 | -1.50993 | 0.072237 |
| GSTP1 | 9.4E-08 | -1.49162 | 0.071042 |
| IFI6 | 0 | -1.47653 | 0.20896 |
| UPP1 | 9.13E-06 | -1.45604 | 0.056114 |
| EIF2AK2 | 2.37E-09 | -1.38809 | 0.10316 |
| TRIM22 | 0 | -1.38411 | 0.175569 |
| TRABD2A | 6.94E-05 | -1.37237 | 0.054522 |
| OXNAD1 | 1.02E-09 | -1.35789 | 0.113105 |
| MX1 | 3.66E-12 | -1.3298 | 0.147565 |
| OAS1 | 1.11E-05 | -1.32775 | 0.069296 |
| OAS2 | 8.48E-06 | -1.31958 | 0.072425 |
| GBP1 | 5.47E-05 | -1.22266 | 0.074384 |
| LPAR6 | 0.000191 | -1.20021 | 0.068792 |
| CISH | 8.89E-06 | -1.19808 | 0.09196 |
| CD96 | 4.91E-07 | -1.18875 | 0.113766 |
| TMEM204 | 8.65E-05 | -1.18853 | 0.076806 |
| LDLRAP1 | 3.97E-08 | -1.13916 | 0.147392 |
| IRF7 | 1.97E-08 | -1.08946 | 0.17145 |
| LINC00861 | 5.24E-09 | -1.08895 | 0.183004 |
| GIMAP5 | 9.42E-05 | -1.08544 | 0.095496 |
| SAMD9L | 0.000918 | -1.07832 | 0.075482 |
| MX2 | 1E-05 | -1.05484 | 0.124984 |

**Table S7. Differentially expressed genes in MTC Reg vs. T Reg**
The top 25 differentially upregulated and downregulated differentially expressed genes (DEGs) in malignant regulatory T cells (MTC Reg) compared to benign regulatory T cells (T Reg) in cutaneous T cell lymphoma (CTCL) patients were ranked by log2 fold change. DEGs with an adjusted p-value (q-value) <0.05, corrected for multiple testing using the Benjamini-Hochberg (FDR) procedure, were selected. The table includes the following columns: q-value (FDR-adjusted p-value), log2fc (log2 fold change), and mean (average expression).

| **gene** | **qval** | **log2fc** | **mean** |
| --- | --- | --- | --- |
| PTPRCAP | 0 | 2.204648 | 0.297684 |
| PTP4A1 | 0 | 2.187884 | 0.120361 |
| HLA-DRB5 | 0 | 2.059033 | 0.922416 |
| IGKV2-30 | 0 | 1.930552 | 0.108779 |
| CD99 | 0 | 1.830564 | 0.203576 |
| IFI44L | 0 | 1.757731 | 0.213809 |
| AKAP17A | 0 | 1.722752 | 0.074434 |
| CDKN1A | 0 | 1.715687 | 0.08055 |
| AC245100.1 | 0 | 1.672851 | 0.09135 |
| SLC25A6 | 0 | 1.651423 | 0.625785 |
| IGHV4-34 | 0 | 1.629368 | 0.115847 |
| IGHV3-21 | 0 | 1.579901 | 0.158601 |
| ATP6V0C | 0 | 1.578001 | 0.26182 |
| P2RY8 | 0 | 1.540341 | 0.114065 |
| IFITM1 | 0 | 1.521891 | 0.487191 |
| EGR1 | 0 | 1.504525 | 0.055531 |
| IGHV3-48 | 0 | 1.500048 | 0.102058 |
| IGLV1-51 | 7.51E-08 | 1.483603 | 0.064132 |
| GTPBP6 | 0 | 1.389999 | 0.061811 |
| IGHV4-39 | 4.56E-12 | 1.379987 | 0.105376 |
| IGLV3-1 | 0 | 1.308325 | 0.1765 |
| COL6A3 | 3.97E-16 | 1.306146 | 0.054172 |
| NR4A1 | 0 | 1.269301 | 0.072652 |
| IFI6 | 0 | 1.265174 | 0.172231 |
| IGKV1-12 | 4.22E-14 | 1.22565 | 0.050584 |
| AC007950.1 | 0 | -283.913 | 0.193011 |
| AL645728.1 | 0 | -283.913 | 0.052531 |
| OSBPL10-AS1 | 2.82E-07 | -5.91548 | 0.087253 |
| ANGPTL1 | 1.24E-06 | -5.44709 | 0.056908 |
| SPON1 | 5.97E-08 | -5.28332 | 0.064837 |
| AC012368.1 | 1.64E-10 | -4.9578 | 0.075087 |
| GPR174 | 1.45E-12 | -4.84262 | 0.085654 |
| TIGD5 | 1.46E-12 | -4.82563 | 0.08586 |
| GPM6B | 1.28E-08 | -4.76959 | 0.052914 |
| AC129492.1 | 1.72E-09 | -4.63221 | 0.05413 |
| PF4 | 3.3E-11 | -4.47036 | 0.061867 |
| LRRC75A | 0 | -4.17371 | 0.10107 |
| U2AF1L5 | 1.49E-11 | -4.0962 | 0.05443 |
| GDF7 | 0 | -4.03266 | 0.087304 |
| CBWD3 | 0 | -4.02501 | 0.118899 |
| GPR82 | 1.65E-11 | -3.97542 | 0.051392 |
| ARL17B | 3.84E-14 | -3.89177 | 0.064146 |
| ANGPT2 | 1.54E-14 | -3.8695 | 0.062575 |
| SLC16A4 | 6.31E-14 | -3.82934 | 0.059069 |
| PLCE1 | 2.28E-12 | -3.81743 | 0.051384 |
| FLCN | 0 | -3.80843 | 0.081171 |
| FAM153C | 1.63E-12 | -3.78865 | 0.051906 |
| C1orf56 | 0 | -3.78207 | 0.268202 |
| HCG11 | 3.97E-16 | -3.77499 | 0.067916 |
| P2RX5-TAX1BP3 | 0 | -3.76127 | 0.102284 |

**Table S8. Differentially expressed genes in B Cells of CTCL patients vs. healthy controls**
The top 25 differentially upregulated and downregulated genes in B cells from patients with cutaneous T cell lymphoma (CTCL), including Sézary syndrome and leukemic mycosis fungoides, compared to healthy controls. Genes were ranked by log2 fold change. Differentially expressed genes (DEGs) with an adjusted p-value (q-value) <0.05, corrected for multiple testing using the Benjamini-Hochberg (FDR) procedure, were selected. The table includes the following columns: q-value (FDR-adjusted p-value), log2fc (log2 fold change), and mean (average expression).

| **gene** | **qval** | **log2fc** | **mean** |
| --- | --- | --- | --- |
| CD99 | 0 | 2.669226 | 0.298811 |
| CSF2RA | 0 | 2.600635 | 0.050871 |
| HLA-DRB5 | 0 | 2.584502 | 0.441027 |
| SCO2 | 0 | 2.498045 | 0.111132 |
| PTP4A1 | 0 | 2.482298 | 0.050776 |
| SLC25A6 | 0 | 2.456743 | 0.585514 |
| HLA-DQA2 | 0 | 2.315161 | 0.090049 |
| IL32 | 0 | 2.254126 | 0.102186 |
| DUSP2 | 0 | 2.092933 | 0.120795 |
| ATP6V0C | 0 | 2.063411 | 0.378556 |
| CCL4 | 0 | 2.02859 | 0.080501 |
| WDR83OS | 0 | 2.026992 | 0.291444 |
| NR4A1 | 0 | 1.973882 | 0.113203 |
| EEF1G | 0 | 1.899744 | 0.175541 |
| CD83 | 0 | 1.858036 | 0.13687 |
| NME2 | 0 | 1.841362 | 0.332602 |
| HLA-DQA1 | 0 | 1.821424 | 0.13639 |
| CCL3 | 0 | 1.8119 | 0.159412 |
| EGR2 | 0 | 1.698165 | 0.069615 |
| RBKS | 0 | 1.68838 | 0.056677 |
| IL1B | 0 | 1.659863 | 0.157601 |
| IFITM1 | 0 | 1.587796 | 0.270852 |
| ATF3 | 0 | 1.509998 | 0.064576 |
| NBPF19 | 0 | 1.479769 | 0.102932 |
| A1BG | 0 | 1.457523 | 0.079872 |
| ALPL | 0 | -283.913 | 0.096514 |
| CD177 | 0 | -283.913 | 0.114852 |
| AC007950.1 | 0 | -8.83432 | 0.44669 |
| CA4 | 3.97E-07 | -7.91638 | 0.062152 |
| DAAM2 | 0 | -6.61694 | 0.168966 |
| CCNJL | 0 | -6.35411 | 0.057331 |
| ARG1 | 0 | -6.29043 | 0.10313 |
| AC129492.1 | 0 | -6.22105 | 0.282921 |
| ZDHHC19 | 0 | -5.99133 | 0.050716 |
| OLAH | 0 | -5.89941 | 0.087222 |
| AMPH | 0 | -5.79501 | 0.300673 |
| IL18RAP | 0 | -5.40586 | 0.071264 |
| IL18R1 | 0 | -5.12678 | 0.098658 |
| MME | 0 | -5.00785 | 0.092502 |
| ITGB3 | 0 | -4.99989 | 0.077409 |
| SH2D4A | 0 | -4.96199 | 0.055308 |
| ADGRG3 | 0 | -4.87806 | 0.082209 |
| ANXA3 | 0 | -4.85379 | 0.086163 |
| MAOA | 0 | -4.83089 | 0.084331 |
| IGHA1 | 0 | -4.55383 | 0.108679 |
| CXCR1 | 0 | -4.51858 | 0.087939 |
| ABLIM3 | 0 | -4.49781 | 0.06519 |
| PTCRA | 0 | -4.118 | 0.053426 |
| HPGD | 0 | -4.00535 | 0.112635 |
| IGKC | 0 | -3.99919 | 0.109335 |

**Table S9. Differentially expressed genes in monocytes of CTCL patients vs. healthy controls**
The top 25 differentially upregulated and downregulated genes in monocytes from cutaneous T cell lymphoma (CTCL) patients versus healthy controls. Genes were ranked by log2 fold change. DEGs with a q-value <0.05, adjusted using the Benjamini-Hochberg (FDR) method, were included. Columns presented are: q-value, log2fc, and mean expression.

| **gene** | **qval** | **log2fc** | **mean** |
| --- | --- | --- | --- |
| LAT | 3.57E-06 | 3.476533 | 0.24838 |
| IL32 | 0 | 2.790161 | 0.778548 |
| IL7R | 1.47E-09 | 2.776181 | 0.351861 |
| CD3D | 2.07E-10 | 2.532662 | 0.367445 |
| CD3E | 8.38E-13 | 2.45468 | 0.461573 |
| CD3G | 5.18E-07 | 2.184682 | 0.215879 |
| CD27 | 4.86E-06 | 1.919135 | 0.180572 |
| PTPRCAP | 5.09E-09 | 1.904221 | 0.302072 |
| CD5 | 0.000466 | 1.853396 | 0.106035 |
| SPOCK2 | 2.47E-05 | 1.793213 | 0.156652 |
| LCK | 1.61E-06 | 1.766795 | 0.205696 |
| AQP3 | 1.76E-06 | 1.734558 | 0.205805 |
| LEF1 | 4.76E-06 | 1.689604 | 0.190336 |
| IFITM1 | 0 | 1.553029 | 0.830668 |
| ID3 | 0.000295 | 1.528046 | 0.12561 |
| CDKN1A | 6.57E-06 | 1.497782 | 0.200072 |
| LIME1 | 4.38E-06 | 1.476242 | 0.2089 |
| CCR7 | 5.47E-06 | 1.403518 | 0.22014 |
| CRIP1 | 0 | 1.390393 | 0.737052 |
| P2RY8 | 0.000568 | 1.381606 | 0.12288 |
| TRIB1 | 0.011744 | 1.367376 | 0.066201 |
| LMNA | 2.5E-07 | 1.359333 | 0.29862 |
| LTB | 3.94E-14 | 1.306232 | 0.661298 |
| HLA-DQA2 | 4.56E-07 | 1.292167 | 0.312265 |
| SYNE2 | 0.000212 | 1.291037 | 0.150994 |
| PRSS57 | 0 | -5.34836 | 0.272078 |
| LAPTM4B | 0 | -4.19166 | 0.157068 |
| MYB | 0 | -3.82549 | 0.201094 |
| ZNRF1 | 0 | -3.32355 | 0.149307 |
| MAP7 | 0 | -3.3156 | 0.111349 |
| FTX | 4.43E-14 | -3.29558 | 0.095588 |
| EVA1B | 0 | -3.13655 | 0.103075 |
| SLC39A3 | 0 | -2.85301 | 0.149135 |
| ATP8B4 | 0 | -2.82859 | 0.124242 |
| MLLT3 | 0 | -2.81654 | 0.171118 |
| SNHG19 | 0 | -2.78746 | 0.105576 |
| FAM117A | 0 | -2.75997 | 0.167 |
| HIST3H2A | 0 | -2.68252 | 0.14264 |
| C11orf74 | 0 | -2.63974 | 0.101671 |
| PDZD8 | 0 | -2.55589 | 0.13589 |
| BEX2 | 0 | -2.51053 | 0.145012 |
| GOLIM4 | 1.48E-14 | -2.48067 | 0.097181 |
| CASP6 | 7.88E-15 | -2.47729 | 0.100462 |
| GCSH | 1.55E-12 | -2.47149 | 0.081898 |
| AGPS | 0 | -2.46832 | 0.149517 |
| GSE1 | 0 | -2.46601 | 0.103186 |
| FHL1 | 7.88E-15 | -2.46499 | 0.100658 |
| SAP30 | 1.74E-11 | -2.39076 | 0.075013 |
| CLEC11A | 0 | -2.39035 | 0.118837 |
| PRKAR2B | 0 | -2.37612 | 0.109186 |

**Table S10. Differentially expressed genes in dendritic cells of CTCL patients vs. healthy controls**
The top 25 differentially upregulated and downregulated DEGs in dendritic cells from cutaneous T cell lymphoma (CTCL) patients versus healthy controls, ranked by log2 fold change. DEGs were selected based on an FDR-adjusted q-value <0.05 using the Benjamini-Hochberg correction. Table columns include: q-value, log2fc, and mean expression.

| **gene** | **qval** | **log2fc** | **mean** |
| --- | --- | --- | --- |
| HLA-DRB5 | 0 | 2.879126 | 0.096005 |
| PTP4A1 | 0 | 2.677696 | 0.091731 |
| PTPRCAP | 0 | 2.556597 | 0.379825 |
| CD99 | 0 | 2.075526 | 0.719643 |
| SLC25A6 | 0 | 2.041345 | 0.501535 |
| IFI44L | 0 | 2.030567 | 0.09364 |
| COL6A3 | 0 | 1.935815 | 0.06132 |
| DIABLO | 0 | 1.917681 | 0.050406 |
| VAMP7 | 0 | 1.876816 | 0.050774 |
| P2RY8 | 0 | 1.867525 | 0.132974 |
| NME2 | 0 | 1.816237 | 0.250667 |
| GTPBP6 | 0 | 1.815905 | 0.080447 |
| NBPF19 | 0 | 1.804227 | 0.058873 |
| AKAP17A | 0 | 1.794085 | 0.076033 |
| EEF1G | 0 | 1.793207 | 0.135608 |
| AC245100.1 | 0 | 1.71499 | 0.050606 |
| ATP6V0C | 0 | 1.710443 | 0.266961 |
| OVCA2 | 0 | 1.59904 | 0.072641 |
| WDR83OS | 0 | 1.559669 | 0.335635 |
| LIME1 | 0 | 1.550847 | 0.124924 |
| HLA-DRB1 | 0 | 1.528766 | 0.233903 |
| KIR2DL3 | 0 | 1.402651 | 0.122237 |
| ALDOA | 0 | 1.278934 | 0.560829 |
| ZC3H11A | 0 | 1.243875 | 0.085019 |
| LMNA | 0 | 1.240459 | 0.065714 |
| TREML1 | 0 | -283.913 | 0.104837 |
| AC007950.1 | 0 | -283.913 | 0.137626 |
| ITGB3 | 0 | -283.913 | 0.058841 |
| ITGA2B | 0 | -6.4419 | 0.118951 |
| GP9 | 2.75E-14 | -5.88716 | 0.06179 |
| PF4V1 | 2.35E-15 | -5.85574 | 0.065876 |
| TUBB1 | 0 | -5.83672 | 0.116852 |
| F13A1 | 5.96E-16 | -5.44667 | 0.051787 |
| NRGN | 0 | -5.18433 | 0.237469 |
| PF4 | 0 | -5.07119 | 0.159652 |
| PPBP | 0 | -4.93258 | 0.274671 |
| PRKAR2B | 0 | -4.64184 | 0.092226 |
| SPARC | 0 | -4.4615 | 0.054347 |
| GNG11 | 0 | -4.3465 | 0.05954 |
| IGHA1 | 0 | -4.11452 | 0.150022 |
| AC129492.1 | 0 | -4.07712 | 0.075484 |
| IGHM | 0 | -4.03221 | 0.061106 |
| IGKC | 0 | -3.79347 | 0.153506 |
| IGFBP2 | 0 | -3.78881 | 0.07984 |
| AL645728.1 | 0 | -3.77998 | 0.062771 |
| PCDHGB6 | 0 | -3.59213 | 0.050939 |
| DOK6 | 0 | -3.58051 | 0.052386 |
| TSIX | 0 | -3.38433 | 0.07231 |
| MIA2 | 0 | -3.29841 | 0.166472 |
| HIPK1-AS1 | 0 | -3.26339 | 0.072775 |

**Table S11. Differentially expressed genes in NK cells of CTCL patients vs. healthy controls**
The top 25 differentially upregulated and downregulated genes in natural killer (NK) cells from cutaneous T cell lymphoma (CTCL) patients compared to healthy controls. Genes were ranked by log2 fold change. Only DEGs with a q-value <0.05 (FDR-adjusted using the Benjamini-Hochberg method) were included. Columns shown are: q-value, log2fc, and mean expression.
